# Experimental Models of Brugada syndrome

**DOI:** 10.3390/ijms20092123

**Published:** 2019-04-29

**Authors:** Franziska Sendfeld, Elisabet Selga, Fabiana S. Scornik, Guillermo J. Pérez, Nicholas L. Mills, Ramon Brugada

**Affiliations:** 1 Scottish Centre for Regenerative Medicine, University of Edinburgh, Edinburgh EH16 4UU, UK; f.sendfeld@gmail.com; 2 BHF/University Centre for Cardiovascular Science, University of Edinburgh, Edinburgh EH16 4TJ, UK; nick.mills@ed.ac.uk; 3 Faculty of Medicine, University of Vic-Central University of Catalonia (UVic-UCC), 08500 Vic, Spain; eselga@gencardio.com; 4 Centro de Investigación Biomédica en Red de Enfermedades Cardiovasculares (CIBERCV), 28029 Madrid, Spain; rbrugada@idibgi.org; 5 Department of Medical Sciences, Faculty of Medicine, Universitat de Girona (UdG), 17071 Girona, Spain; 6 Usher Institute of Population Health Sciences and Informatics, Edinburgh EH16 4SU, UK; 7 Cardiovascular Genetics Centre, Institut d’Investigació Biomèdica de Girona (IDIBGI). Hospital Josep Trueta, 17007 Girona, Spain

**Keywords:** Brugada syndrome, *SCN5A*, model systems, induced pluripotent stem cells (iPS)

## Abstract

Brugada syndrome is an inherited, rare cardiac arrhythmogenic disease, associated with sudden cardiac death. It accounts for up to 20% of sudden deaths in patients without structural cardiac abnormalities. The majority of mutations involve the cardiac sodium channel gene *SCN5A* and give rise to classical abnormal electrocardiogram with ST segment elevation in the right precordial leads V1 to V3 and a predisposition to ventricular fibrillation. The pathophysiological mechanisms of Brugada syndrome have been investigated using model systems including transgenic mice, canine heart preparations, and expression systems to study different *SCN5A* mutations. These models have a number of limitations. The recent development of pluripotent stem cell technology creates an opportunity to study cardiomyocytes derived from patients and healthy individuals. To date, only a few studies have been done using Brugada syndrome patient-specific iPS-CM, which have provided novel insights into the mechanisms and pathophysiology of Brugada syndrome. This review provides an evaluation of the strengths and limitations of each of these model systems and summarizes the key mechanisms that have been identified to date.

## 1. Introduction

**Figure 1:**
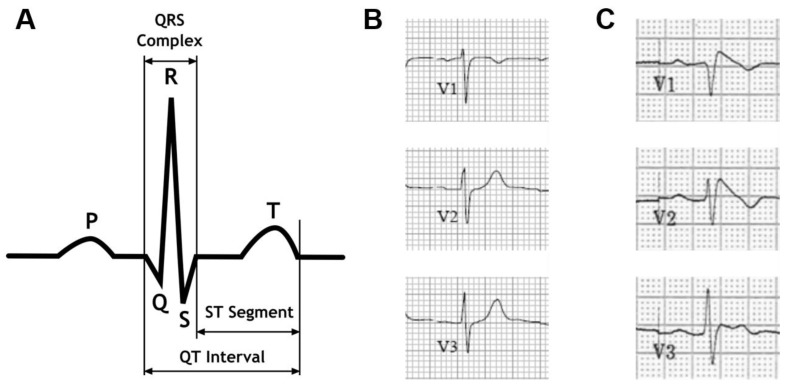
The electrocardiogram in the Brugada syndrome. (**A**) Schematic diagram of a normal electrocardiographic complex representing sinus rhythm. (**B**) Precordial leads V1-V3 from a normal electrocardiogram. (**C**) Precordial leads V1-V3 from a patient with Brugada syndrome.

The Brugada syndrome, first described by the Brugada brothers in 1992 [1], is a life-threatening arrhythmogenic disease characterized by an abnormal electrocardiogram (Figure 1) with ST segment elevation in the right precordial leads V1 to V3 and right bundle-branch block. It is responsible for 4 to 12% of sudden cardiac deaths in the general population. The Brugada syndrome is an inherited disease typically transmitted in an autosomal-dominant manner with incomplete penetrance. Patients often present with symptoms of ventricular tachycardia, bradycardia, or atrial-ventricular (AV) node conduction disorder. These symptoms are more often present in males than in females.

Brugada syndrome was initially proposed as a primary electrical disease in structurally normal heart [2,3]. However structural abnormalities found in the right ventricle outflow tract (RVOT) of patients suggest that Brugada syndrome may belong to a larger spectrum of right ventricular cardiomyopathie [4].

To date, the implantation of a cardioverter defibrillator is the only effective treatment for the conditio [5,6]. The clinical phenotype and its manifestations have been reviewed in detail elsewher [6]. 

Genetic mutations are identified in 11–28% of patients with Brugada syndrome, of which the majority (>90%) are located in the *SCN5A* gene [2]. This gene encodes the alpha-subunit of the cardiac sodium channel Na_v_1.5, which is responsible for the sodium inward current (I_Na_). Mutations in other channels, or channel regulatory proteins, have also been linked to the Brugada syndrome. So far, more than 20 genes have been associated with this diseas [7]. These include genes encoding for the L-type calcium channels Cav1.2 (*CACNA1c*) and Cavß2b (*CACNB2b*) [8,9], glycerol-3-phosphate dehydrogenase 1-like enzyme (*GPD1L*) [10], β-subunits of the sodium channel (*SCN1* [11], *SCN2* [12], *SCN3* [13]) and MiRP2 (*KCNE3*) [14], among others. However, disease-gene associations were recently re-evaluated by an expert consortium of the Clinical Genomic Resource (ClinGen). This evidence-based gene validation for Brugada syndrome indicated that 20 of 21 genes lack sufficient evidence to support causality for Brugada syndrome. Regarding clinical validity, only the *SCN5A* gene was classified as demonstrating definitive evidence as a cause for Brugada syndrome [15]. Variants in other genes may still contribute to susceptibility to Brugada syndrome, suggesting that this disease, initially considered as autosomal dominant monogenic, could actually be multigenic. In the present review, we will focus on models used to study Brugada syndrome related to *SCN5A* mutations, with limited mention of other genes.

Whilst the role of loss of function of the Nav1.5 channel in Brugada syndrome is undisputed, there is disagreement as to the mechanism underlying the pathophysiology of this disease. Two main hypotheses have been proposed: The “repolarization hypothesis” based on the transmural dispersion of repolarization in the right ventricle (RV) between the endocardium and epicardiu [16]; and the “depolarization hypothesis” based on RV conduction slowing and presence of subtle structural abnormalitie [17]. Arguments for and against either hypothesis have been extensively reviewed and discusse [17,18].

**Figure 2:**
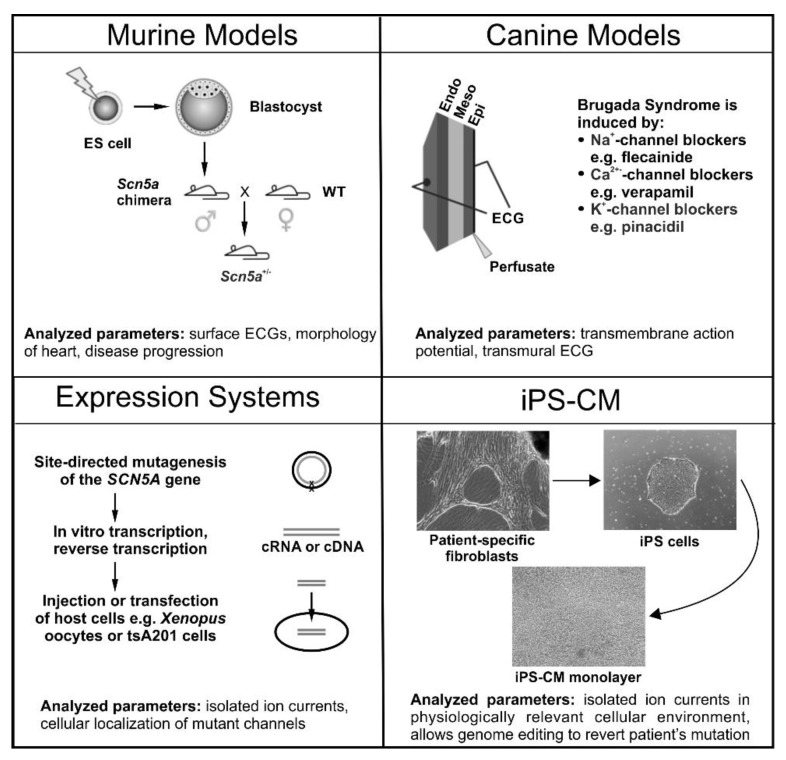
A Schematic overview of the experimental models predominately used for studying Brugada syndrome. Murine Models: Generated through targeted disruption of *Scn5a*. Manipulated ES cells are transfected into blastocysts to give rise to a male chimera. Crossing with wild type females results in heterozygous offspring (*Scn5a*^+/−^). Canine Models: Wedges of canine left or right ventricles are perfused arterially. Brugada syndrome is drug-induced with Na+-channel blockers (e.g., pilsicainide), Ca^2+^-channel blockers (e.g., verapamil) or K+-channel openers (e.g., pinacidil), which are administered either by themselves or in combination. Expression Systems: Families displaying symptoms of Brugada syndrome are screened for mutations using direct sequencing with a candidate gene approach. Relevant mutations are then introduced into vectors carrying the gene of interest using site-directed mutagenesis, and subsequently transfected into a host cell (e.g., tsA201 cells) or in vitro transcribed and injected into Xenopus oocytes. Cardiomyocytes from Induced Pluripotent Stem (iPS) Cells: Dermal fibroblasts are isolated, cultured and transfected with the pluripotency factors Oct3/4, Klf4, Sox2, and c-Myc. Transfected fibroblasts are then cultured in human embryonic stem cell selection media until colonies of iPS cells are detected. Selected iPS cells then undergo cardiomyocyte differentiation using either unguided (spontaneous) differentiation, guided differentiation (cytokines, growth factors) or through co-culture. The parameters analyzed and key findings for each model system are summarized at the bottom of each panel.

Case studies have contributed to understanding the mechanisms underlying Brugada syndrome as well as to improve patient diagnosis and treatmen [3,19,20,21]. Although the human heart represents the gold standard for the study of Brugada syndrome inherent methodological and ethical limitations impose the need to rely on animal and cellular studies. Different models have been used to simulate this disease (Figure 2), including transgenic mice, canine heart preparations, transgenic pork, expression of mutant *SCN5A* in different cellular models and induced pluripotent stem cell-derived cardiomyocytes (iPS-CM).

This review aims to analyze the experimental systems most commonly used to model Brugada syndrome. Knowledge gained from studies performed using the different models has contributed to our current understanding of mechanisms involved in Brugada syndrome. Here we discuss their major accomplishments as well as their strengths and limitations. 

## 2. Murine Models

Two approaches have been conceived. In one, the murine equivalent of the human *SCN5A* gene (*Scn5a*) is knocked out to simulate a generic Brugada syndrome phenotype. In the other, a specific mutation found in a family with Brugada syndrome is introduced to replicate this particular phenotype.

### 2.1. Scn5a Heterozygous Knockout Mice

In 2002, Papadatos et al. [22] generated a mouse model for arrhythmias through targeted disruption of *Scn5a* (Figure 2). Homozygous knockout mice exhibited embryonic lethality due to structural abnormalities of the heart. Heterozygous knockout mice (*Scn5a*^+/−^) were indistinguishable from their wild type (WT) littermates with regards to weight, heart/body weight ratio and cardiac morphology. The electrocardiogram of *Scn5a*^+/−^ mice revealed slowed conduction compared to WT mice, but a normal QT-interval. Whole-cell patch-clamp recordings confirmed these findings and demonstrated a ~50% reduction in Na^+^ conductance, explaining impaired action potential propagation, conduction block, re-entrant arrhythmias and ventricular tachycardia in this model. However, *Scn5a*^+/−^ mice exhibited several cardiac phenotypes as opposed to one strictly representative of the Brugada syndrome. Phenotypic heterogeneity in *Scn5a*^+/-^ mice as well as in patients carrying *SCN5A* mutations is considerable, even resulting in some symptom-free mutation carriers. This heterogeneity could at least in part be explained by varying levels of Na_v_1.5 protein expression, with the severity of the phenotype correlating with expression levels of Na_v_1.5 [23].

The mouse model described in this initial report was subsequently used to investigate how far it recapitulates clinical phenotypes observed in Brugada syndrome patients, to assess drug response, and to examine the underlying mechanisms of those phenotypes observed. Age and sex-related factors in disease progression such as an increased risk of cardiac-related mortality, and AV node conduction disease and bradycardia in old males have been replicate [24,25,26,27,28].

Studies aiming to recapitulate the response of Brugada syndrome patients to pro- and anti-arrhythmic drugs showed that in Langendorff-perfused *Scn5a*^+/−^ hearts, flecainide increased RV transmural gradients while quinidine decreased them, in line with their respective pro- and antiarrhythmic effect [29,30,31]. Anesthetized WT and *Scn5a*^+/−^ mice were also used to record electrocardiograms which revealed that ventricular arrhythmias, ST-segment elevation, transmural repolarization gradients and abnormal conduction present in these animals were accentuated by treatment with flecainid [32]. 

Martin et al. [33] used the mouse model to investigate molecular and functional differences between RV and left ventricle (LV) in the *Scn5a*^+/−^ compared to the WT heart. Na_v_1.5 mRNA and protein expression were lower in *Scn5a*^+/−^ than in wild-type (WT) animals, with a further reduction in the RV compared to the LV. Action potential upstroke velocity and maximum Na+ current density were correspondingly decreased in *Scn5a*^+/−^, with a further reduction in the RV compared to the LV. The initial reduction in Na_v_1.5 expression was confirmed by Zhang et al. [34]. These authors also investigated the characteristics of the ventricular effective refractory periods, conduction velocities and dispersion in conduction direction in relation to this decreased Na_v_1.5 expression. This was further related to the appearance of fibrosis. Recently, this has been thoroughly reviewed by Huan [35].

Although many of the results obtained using the *Scn5a*^+/−^ mice support the depolarization hypothesis, some contradictions among the above-mentioned studies (editorialized by Tse et al. [36,37]) complicate their interpretation to reach a clear conclusion on the mechanism leading to arrhythmogenesis in Brugada syndrome. 

Interestingly, a recent study by Kelly et al. [38] showed that normal structural heterogeneities present in the RV are sufficient to explain the susceptibility to arrhythmias when sodium current is diminished. Using optical mapping and two-photon microscopy in isolated-perfused mouse hearts, this state-of-the-art study strengthens the claim that the mouse model can faithfully recapitulate the Brugada syndrome phenotype, especially when a mutation in *SCN5A* is present. 

### 2.2. Scn5a-1798insD Knock-in Mice

One of these mutation-specific mouse models was developed by Remme and coworker [39], who generated a mouse strain carrying the murine equivalent of the human *SCN5A*-1795insD mutation. This mutation had been identified in different members of a Dutch family, who display overlap symptoms of Long QT syndrome 3 (LQT3), Brugada syndrome and progressive cardiac conduction defect [40]. Mice carrying the *Scn5a*-1798insD mutation indeed showed a similar phenotype demonstrating bradycardia, QT prolongation, and right ventricular conduction slowing, confirming that a single mutation was sufficient to cause the overlap syndrome. Interestingly, the severity of the conduction defect caused by the *Scn5a*-1798insD/+ was shown to be strain dependen [41]. This correlated with a lower β4 subunit expression in ventricular tissue resulting in a larger loss of function of Na_v_1.5 current in isolated myocyte [39]. 

### 2.3. Strengths and Limitations of Mouse Models

The advantage of using a model organism rather than a single cell expression system is that a mutation of interest can be investigated in its physiological environment and during different developmental stages. The heterozygous knockout of *Scn5a* in mice generated a mouse model that replicates many features associated with the Brugada syndrome including sex-dependent disease progression and drug response. Both depolarization and hypothesis were supported by evidence provided by this model. Thus, although it has not delivered a conclusive answer regarding the mechanism underlying arrhythmias in Na_v_1.5 haploinsufficient animals, this model has proved valuable for exploring the underlying cause of ventricular tachycardia in patients.

Still, there are some limitations when extrapolating the repolarization findings obtained with the mouse model to the human heart. Besides the obvious difference in size and faster beating rate (four to eight times faster), they present differences in their ion channel patterns compared to the human heart (e.g.,: TTX-sensitive sodium channels transcript levels decreased with increasing heart size) [42]. This different expression profile results in different action potential characteristics and pharmacology.

In addition, engineering mice with patient-specific Brugada syndrome mutations is costly. However, as shown by the recent study by Kelly et al. [38], it is a very valuable model to investigate the interplay between structural and ionic factors in arrhythmogenesis, which cannot be investigated in most of the other available models.

## 3. Porcine Model

Despite the electrophysiological similarities between the porcine and human heart, the expansiveness of this model has limited its use in the research of Brugada syndrome. A work by Park and collaborators used hearts from pigs carrying the porcine equivalent of the human nonsense mutation *SCN5A*-E555X [43]. This [44] mutation had previously been identified in a patient with Brugada syndrome. They reported that hearts from the modified animals showed no structural abnormalities, but they displayed conduction slowing and increased susceptibility to ventricular arrhythmias, compared with wild type animals. These animals did not display a Brugada syndrome ECG pattern, their hearts were rhythmically unstable and manifested spontaneous or inducible ventricular fibrillation. Whether the lack of a clear Brugada syndrome ECG was due to the absence of fibrosis in the modified pig heart remains unresolved.

## 4. Canine Models

Canine models have been widely used for the study of cardiac electrophysiology. Almost all data obtained from the canine model for Brugada syndrome were acquired using arterially perfused wedges of canine left or right ventricles rather than a whole heart. Perfused wedges are treated with drugs to induce the Brugada syndrome phenotyp [43] (Figure 2). Different drugs have been used to that end, all of which are either Na+ channel blockers (e.g., pilsicainid [45]), Ca2+ channel blockers (e.g., verapami [46]) or K+ channel openers (e.g., pinacidi [47]) and are administered either alone or in combination. These induced systems have been exploited to investigate the underlying electrophysiological cause of the different abnormalities that can be usually observed on the Brugada syndrome electrocardiogram. Canine heart experiments have been key in understanding several arrhythmia-related ECG landmarks, such as ST-segment elevation, the J-wave/Osborne wave, and T-wave alternans and ventricular tachycardia or fibrillation.

### 4.1. Use of Canine Models to Uncover the Biophysical Causes of the Alterations in the Electrocardiogram

#### 4.1.1. The J-wave/Osborne Wave

The J-wave, also called Osborne wave, is a positive deflection located between the QRS and ST-segment on the electrocardiogram (Figure 1) and is found in patients at risk of ventricular fibrillation such as those with Brugada syndrome. Drug-induced canine heart preparations were first proposed as a suitable model system for the Brugada syndrome in 1996 when Yan and Antzelevitch investigated the underlying cause of the J-wav [43]. Transmembrane action potential recordings suggested a voltage gradient across the ventricular wall. Similar results could be achieved by cooling the right ventricular outflow tract in sit [48], providing a possible in vivo model.

#### 4.1.2. The ST-Segment Elevation

ST-segment elevation is the most striking feature and the primary criteria used for diagnosis of a patient with classical Brugada syndrome. Several groups have pursued investigations to define the electrophysiological alterations that result in ST-segment elevation during ventricular repolarization. Yan and Antzelevitch used flecainide and acetylcholine to induce Brugada syndrome in a canine model. This approach allowed them to propose that ST-segment elevation is caused by the same transmural voltage gradient across the right ventricular wall that causes the J-wav [16,43]. The transmural voltage gradient is caused by depression or loss of the epicardial action potential dome. Their results also indicated that phase 2 reentry (P2R) could cause extra-systolic activity in the right ventricular wall which could serve as a substrate for ventricular arrhythmias. ST-segment elevation had previously been linked to electrical heterogeneity in the recovery phase, a substrate for P2 [45]. Thus, this model has provided evidence showing that transmural dispersion of ventricular repolarization leads to ST-segment elevation. Further experiments have shown that repolarization abnormalities associated with ST-segment elevation are located in the right ventricular outflow tract [49,50]. This last study showed that epicardial heterogeneity of action potential induced transmural heterogeneity of action potential, contributing to P2R and ventricular tachycardia. 

#### 4.1.3. T-Wave Alternans and Ventricular Tachycardia or Fibrillation

Another feature of the Brugada phenotype associated with loss of the action potential dome is T-wave alternans. This arises due to beat-to-beat variation in amplitude, polarity, and morphology of the T-wave, and has been associated with an increased risk of ventricular arrhythmia. Morita et al. [51] were the first to describe its electrophysiological foundation and showed that it is at least in part caused by the same increased dispersion of repolarization that causes ST-segment elevation. Later, Fish and Antzelevitch [52] showed that T-wave alternans share another feature with ST-segment elevation. The results from a right ventricular wedge preparation perfused with verapamil, a sodium and calcium channel blocker, indicated that T-wave alternans could be caused not only by the loss of the action potential dome but also by a concealed P2R which both lead to transmural dispersion of repolarization. Whilst mutations in both the cardiac sodium- or calcium-channel may cause Brugada syndrome, there are phenotypic differences between the [51]. The interpretation of data generated from model systems using a combined sodium-calcium channel block approach is therefore challenging.

### 4.2. Use of Canine Models to Assess the Cause for Sex Differences

One interesting aspect about Brugada syndrome is that even though mutations causing the disease are equally inherited by males and females, males are 8-10 times more likely to develop symptom [52]. Recordings performed in cardiomyocytes isolated from canine epicardium have shown a smaller transient outward potassium current (Ito) density in females compared to males. This reduced Ito could protect females from developing Brugada syndrome, but make them more prone to progressive conduction problems when inward currents like I_Na_ are compromise [53].

### 4.3. Use of Canine Models to Identify Markers of Risk and to Improve Treatment

Even though discovering the mechanisms that lead to ventricular arrhythmias is important, the ultimate goal remains to apply the gathered knowledge to improve methods for diagnosis, prediction of high-risk patients and treatment. Investigations of conduction abnormalities, such as right bundle branch block, led to the discovery of fragmented QRS as a new marker for Brugada patients with a high risk of syncope and developing spontaneous ventricular fibrillatio [54]. Fragmented QRS are conduction abnormalities within the QRS complex and can manifest as multiple spikes in the QRS segment. In terms of treatment, most strategies aim at preventing sudden death caused by ventricular arrhythmias rather than treating the underlying cause, i.e., loss of sodium channel function. Using the canine model, researchers were able to show that a more focused application of radiofrequency catheter ablation (RFA) to the epicardium might be more efficient in eradicating ventricular tachycardia in Brugada syndrome patient [55]. A more recent study showed that the beneficial effects of RFA are based on destroying the cells with the most prominent action potential notch in the epicardial surface. The authors conclude that RFA eliminates sites of abnormal repolarization and therefore the substrate for ventricular tachycardia and ventricular fibrillatio [56]. These examples illustrate how the canine model system could help not only to find new, easy to measure, markers for high-risk patients but also suggest possible improvements for established treatments.

### 4.4. Strengths and Limitations of the Canine Model

Canine preparations have proven to be a useful system for modeling Brugada syndrome. Not only have they linked J-waves, ST segment elevation and T-wave alternans to a depression of the action potential dome, they were also instrumental in determining that a lower I_to_ density in females compared to males, could protect females from developing Brugada syndrome. In addition, the canine model helped reveal fragmented QRS as a new marker for high-risk patients and suggested a novel method of using radiofrequency ablation in Brugada syndrome patients. The main advantage of using the heart wedge preparation is that cells in epi- and endocardium can be investigated preserving their structural organization within the heart. 

An apparent limitation of the canine coronary perfused wedge preparation is that it does not include the RVOT making it difficult to test the hypothesis of depolarization as the underlying mechanism for arrhythmogenesis in Brugada syndrome patients. Nevertheless, by using NS5806 in conjunctions with verapamil on this model to mimic the Brugada syndrome phenotype, Szél and Antzelevitc [20] showed that late potentials and fractionated electrogram arise from heterogeneities in the epicardial action potential. They also showed that high-frequency spikes appear as a result of concealed phase-2-reentry. The authors argue that no primary conduction delay was observed as the cause of the Brugada syndrome ECG phenotype or late potential or fractionated electrogram activity. This evidence supports the hypothesis that primary repolarization defects are responsible for the Brugada syndrome phenotype.

Still, the most obvious limitation of this system is that the Brugada syndrome phenotype is drug-induced. Combinations of sodium and calcium channel blockers and/or potassium-channel openers have been used, even though the majority of patients only carry one mutation. Experiments have also shown subtle differences in phenotypes depending on which channel is bearing the mutation. Because the Brugada syndrome phenotype in this model is drug-induced, drug screening is limited not only due to possible interactions with the channel blocking agent but also because mutations underlying Brugada syndrome may be more complex than simple loss-of-function modeled by a drug-induced Brugada syndrome phenotype. Unfortunately, it also does not allow for investigations of either atypical Brugada syndrome patients with no mutation but a Brugada phenotype, or vice versa. 

## 5. Expression Systems

Experiments using expression systems aim at deciphering the biophysical properties of both, wild type and mutant ion channels in single cells. This may help in understanding the molecular mechanisms underlying Brugada syndrome. Plasmids for the expression of genes carrying mutations identified in Brugada syndrome patients are typically transfected into human embryonal kidney, SV40 transformed (tsA201) or Chinese hamster ovary (CHO) cells. In addition, frog oocytes have been widely used for heterologous expression of ion channels (Figure 2). Xenopus laevis oocytes are large and cheap to maintain and manipulate, both of which make them a desirable tool for electrophysiological experiments. tsA201 are the traditional gold standard for ion channel characterization in mammalian single cells. Employing expression systems, investigators have been able to study the biophysical consequences of Brugada syndrome-associated mutations and the effects of external factors on sodium current. 

### 5.1. Use of Expression Systems to Assess the Biophysical Consequences of Brugada Syndrome-Associated Mutations

Mutations in *SCN5A* and other genes associated with Brugada syndrome have been studied using expression systems. In general, these studies revealed a loss of function of sodium channels. Over the decades, the benchmark technique to investigate the nature of mutation-induced loss of function has been the combined approach of patch clamp experiments and heterologous expression system. This approach is uniquely suited for detailed analysis of channels’ biophysical properties. This type of experiments revealed that loss of sodium channel function may result from reduced current density, positive shifts in the voltage dependence of activation or negative shift in steady-state inactivation. Loss of function can also result from acceleration of inactivation kinetics. In addition, once the channel enters the inactivated state, it needs time to return to a state that permits its activation. This time, measured as the recovery from inactivation, is directly related to the refractory period. Thus, changes in this recovery time caused by a mutation may create a substrate for arrhythmogenicity. 

**Figure 3:**
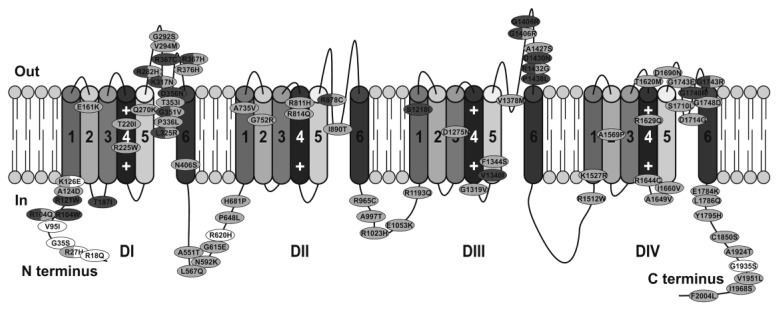
Na_v_1.5 channel scheme showing the location of mutations in *SCN5A* identified in patients with the Brugada syndrome. Only missense mutations that have been functionally characterized using expression systems are displayed. Nonsense and insertion or deletion mutations are not shown. Mutations found to cause a complete loss of I_Na_ are dark grey, those reported to reduce I_Na_ and/or alter Na_v_1.5 properties are light grey, and white indicates no changes in channel properties. Mutations for which two different types of alterations have been described are displayed in both colors.

Since the focus of this review is to discuss the strengths and limitations of model systems, only a few selected studies will be used to highlight the main mechanisms through which mutations can cause Brugada syndrome. A comprehensive list of *SCN5A* mutations studied in heterologous expression systems, identified in Brugada syndrome patients, and their functional consequences are provided in Supplementary Table. A schematic representation of the location of these mutations within Na_v_1.5 is illustrated in Figure 3. A systematic pathogenicity analysis of all *SCN5A* Brugada syndrome variants has been recently reported by Denham et al. [57]. 

#### 5.1.1. Sodium Current Density

Around one-third of the Brugada syndrome-associated mutations in *SCN5A* that have been functionally characterized cause a partial reduction in sodium current density, typically estimated from peak I_Na_ (Supplemental Table). This reduction may arise from defects in channel trafficking or non-conducting channels. Trafficking defects and truncated proteins have both been linked to loss of function of Na_v_1.5 in Brugada syndrome patients. Depending on the mutation, expression in Xenopus oocytes or tsA201 cells can give rise to contradictory observations. One such example is R1432G, which completely abolished functional expression of sodium channels in tsA201 cells while producing slightly reduced sodium current when expressed in Xenopus oocytes. Mutant channels in tSA201 cells were shown to be localized in the endoplasmic reticulum suggesting that the mutation prevents the transport of Na_v_1.5 to the cell surfac [58]. This would be a potential mechanism for Brugada syndrome in patients with this mutation. 

Besides disruption of protein transport to the cell membrane, mutations that result in complete loss of Na_v_1.5 current may arise from defects in protein expression. Some mutations found in Brugada patients, such as E473X and N1774delT, produce truncated proteins unable to generate detectable sodium curren [59]. When these mutant channels were heterozygously expressed with wild type channels, a 50% reduction of sodium current was detected. This reduction, together with the absence of effects on activation or inactivation properties or on current kinetics, indicated that only the wild type channels were functiona [60]. 

#### 5.1.2. Voltage Dependence of Activation and Steady State Inactivation

The reduction in peak I_Na_ can be accompanied by alterations in sodium channel properties. For example, the mutation *SCN5A*-1795insD produced a negative shift of the steady-state inactivation and positive shift of the steady-state activation curve [61,62]. Another example was shown in our work with the Na_v_1.5 mutation I890T. This mutation caused a reduction of current density together with a positive shift of the activation curve, accentuating the loss of functio [63].

#### 5.1.3. Inactivation Kinetics

Mutations can alter the speed of inactivation resulting in accelerated or decelerated inactivatio [64,65]. Whereas a faster current decay reduces the net sodium current, a feature linked to Brugada syndrome, slower current decay is generally associated with LQT3. However, Brugada syndrome-associated mutations can also produce sodium currents with slower decay in combination with reduced peak current or, with altered activation and/or inactivation dependenc [65,66,67].

#### 5.1.4. Recovery from Inactivation

Lastly, prolonged recovery from inactivation could lead to arrhythmias in Brugada patient [68]. In a study of the Na_v_1.5 mutation 1795insD expressed in tsA201 cells, Veldkamp and collaborators proposed that some mutant channels enter an intermediate state of inactivation from which they recover slower than wild type channel [69]. This mechanism, although already known, had not been previously associated with Brugada syndrome. Other mutations were shown to have similar effects. A lengthening in the recovery from inactivation time was also reported for the Na_v_1.5 mutation T1620M. These results were however restricted to expression in tsA201 cells, whereas Xenopus oocytes transfected with the same mutation showed the opposite phenotyp [70]. 

#### 5.1.5. Other Genes Associated with Brugada Syndrome

Although mutations have been identified in genes other than *SCN5A*, only a few have been functionally studied. Mutations in *SCN1* [11], *SCN2* [12], *SCN3* [13] and *GPD1* [10] have been shown to cause a reduction in sodium current, in some cases due to disruption in trafficking of Nav1.5 to the cells surfac [13]. Also, a mutated MiRP2 has been reported to trigger an increased Ito intensit [14]. Two mechanisms have been associated with mutated *CACNA1c* or *CACNB2b* found in Brugada patients, and while both result in loss of function, one is caused by reduction of L-type Ca^2+^-current, and the other one triggers accelerated inactivation of Ca^2+^ curren [9].

### 5.2. Strengths and Limitations of Expression Systems

For a long time, expression systems were the only Brugada syndrome model that permitted investigation of the molecular mechanism of mutations found in patients rather than looking at manifestations of generic *SCN5A* knockout or drug-induced Na_v_1.5 block. Cell-based model systems are inexpensive and relatively easy to maintain and manipulate. Although multiple genes have been associated with Brugada syndrome, the genetic cause for two-thirds of all patients remains unidentified. The limitation of this model system is its restriction to the examination of known genetic causes. It also gives no insight into the heterogeneity of phenotypes between family members carrying the same mutation. One intriguing issue is the contradictory results from the expression of mutant channels in different cell lines. Even though both tsA201 cells and Xenopus oocytes expressing Na_v_1.5 are able to generate sodium currents, they are morphologically and functionally very different. This could explain the discrepant results obtained when analyzing the effects of one particular mutation using both cellular systems. More importantly, it is irrefutable that neither of these two cell models closely resembles human cardiomyocytes. Nevertheless, expression systems can still be very informative on the possible pathogenic effect of individual mutations.

## 6. Induced Pluripotent Stem Cell-Derived Cardiomyocytes (iPS-CM)

Yamanaka and colleague [71] provided an alternative for ethically difficult human embryonic stem cell research that has transformed disease modeling. Yamanaka and others demonstrated that somatic cells can be reprogrammed by the introduction of a number of transcription factors involved in pluripotency and proliferatio [44,72]. The resulting reprogrammed cells are almost indistinguishable from embryonic stem cells in morphology, surface markers, gene expression and ability to differentiate into cells from all three germ layers. The great advantage of this technique is that cells carrying the desired genetic profile can be obtained directly from the patient, reprogrammed into iPS cells and differentiated to the specific cell type that is affected by the disease, e.g., cardiomyocytes (iPS CM). This technique allows investigators to study the mutant proteins in their native environment, which includes modulatory proteins. 

To date, independent studies have shown that iPSC-CMs can effectively recapitulate inherited arrhythmia syndromes at the cellular level. Several cardiac disorders including LQT1 [73], LQT2 [74], CPV [75] and Timothy syndrom [76] have been modelled using the iPS cell approach. The first Brugada syndrome mutation studied in iPS-CM was the *SCN5A*_1795insD mutatio [40]. This mutation causes a Brugada /LQT3 overlap syndrome that had previously been studied in both a mouse model and cell expression systems. These iPS cell-derived cardiomyocytes recapitulated the decrease in peak I_Na_ and persistent I_Na_ associated with Brugada syndrome and LQT3 respectively.

Despite being a relatively recent experimental approach, several research groups have already used iPS-CM derived from Brugada syndrome patients to explore novel therapeutic tools and to study basic mechanisms of the disease. The following paragraphs summarize the major findings of these studies.

Kosmidis et al. [77] explored the therapeutic potential of drugs that induce translational readthrough of premature stop codons to reverse the effect this type of mutations in *SCN5A*. The patient-derived iPS-CM used in this study carried the Na_v_1.5 mutations R1638X or W156X, which cause a loss of sodium channel function. The study concluded that neither gentamicin nor PTC124 were effective in reversing the effect of either mutation. These drugs, however, had been proven effective in restoring protein expression of other mutations. Thus, this work corroborates the use of patient-derived iPS-CM as a unique model to study *SCN5A* therapeutic tools for particular nonsense mutations associated with Brugada syndrome.

A great advantage of iPS cells is that they offer the possibility of genome editing. This was used by Liang et al. [78] in a study in which they compared sodium currents, action potentials and calcium dynamics in iPS-CM derived from two Brugada syndrome patients and two healthy control. The authors reported a reduced sodium current, increased triggered action potential activity, and abnormal Ca^2+^ transients in the Brugada syndrome patient-derived cells in one of the patient-derived iPS cell lines the *SCN5A* mutation (c.4190delA) was edited using the CRISPR/Cas9-mediated genome editing technology. Reversion of the mutation to wild-type normalized the electrical properties of the patient-derived cells, indicating that the mutation was sufficient to cause the defects. This study showed that the combined approach of patient-specific iPS-CM and CRISPR-Cas9 genome editing can be valuable to test genotype-phenotype associations in Brugada syndrome. 

As 70% of Brugada syndrome patients remain without an identified mutation, iPS-CM provide the opportunity to study the cellular mechanisms of disease in those patients and provide clues regarding candidate genes. Interestingly, a thorough study by Veerman et al. [79] using iPS-CM derived from three Brugada syndrome patients without a known coding mutation showed no differences in sodium currents between patients and control-derived cells. These authors concluded that cellular electrophysiological changes may not participate in generating the clinical phenotype in these patients. This could imply that the underlying mechanism for these patients’ phenotype is not caused by defects in ion channels or their modulatory subunits but may instead involve the development of structural changes like fibrosis in the RV. 

Similar results were found by Miller et al. [80]. These authors studied Brugada syndrome-derived iPS-CM from patients with no identified pathogenic mutations. Electrophysiological properties of these cells were indistinguishable from control-derived iPS-CM. Although this study demonstrated that iPS-CM were suitable to test the blocking effect of ajmaline on both depolarization and repolarization, it did not find any difference of this block between Brugada syndrome and control-derived cells. 

One of the main disadvantages of iPS-CM as a model for arrhythmogenic diseases is their immature phenotype. While this seems not to be a major problem for studying most of the cardiomyocytes’ currents, it poses constrains on the study of action potential [81]. 

To overcome this problem, Ma et al. [82] used the dynamic clamp technique to impose the absent inward rectifier current (IK1) onto iPS-CM to achieve a more mature functional phenotype. They studied patient-specific iPS-CM carrying a compound Brugada syndrome mutation (p.A226V and p.R1629X), and a mild Brugada syndrome *SCN5A* mutation (T1620M) in iPS-CM commercially available. They found that the effects of the compound mutation caused a 75% loss of sodium current compared to iPS-CM derived from a non-carrier sibling. This decrease in current correlated with a reduction of the maximum upstroke velocity and action potential amplitude. In addition, they observed early repolarization (shortening of action potential duration, APD) in 25% of the patient-specific derived cells paced at 0.1 Hz. Conversely, they found that iPS-CM with no reduction in sodium current, including controls and iPS-CM with the mild mutation, had normal action potential properties. This study also compared I_to_ levels at different pacing frequencies and found that iPS-CM from Brugada syndrome and control-derived cells were equally heterogeneous regarding this current. The authors used the I_to_ blocker 4-AP to restore APD in the Brugada syndrome patient-derived iPS-CM that showed a reduction in APD to demonstrate the contribution of this current to early repolarization. Thus, this model showed that a coordinated role of I_to_ and loss of sodium current induces arrhythmogenic APD changes as it had been previously demonstrated in other Brugada syndrome models. 

Our laboratory has been using patient-specific iPS-CM to study *SCN5A* mutations related to Brugada syndrome. Our results showed that iPS-CM from a Brugada syndrome patient bearing the mutation p.R367H recapitulated the loss of function of Na_v_1.5 associated with the disease. More interestingly, we observed that in addition to decreasing sodium current, this mutation affected both, current activation and inactivation voltage-dependence, and caused a faster recovery from inactivation. These changes were surprising for a mutation previously described as non-conducting. Interestingly, when the channel was expressed in heterozygosity in tSA201 cells, only the reduction in current density observed in iPS-CM was reproduce [83]. A possible explanation for this is that Na_v_1.5 regulatory proteins that affect channel activity and that are present in iPS-CM but absent in tSA201 cells. 

### Strengths and Limitations of iPS-CM

The iPS cell-based model system has great strengths, especially compared to the expression systems, but also has its limitations. One of its main advantages is that patient-specific iPS-CM not only carry the patient’s exact genetic background but also reproduce the cardiomyocytes’ particular properties. Considering haploinsufficiency of Na_v_1.5 as a key mechanism in Brugada syndrome, patient-derived iPS-CM offer a unique advantage since the transcriptional activity of these cells occurs free of experimental artifacts inherent to heterologous expression systems. Thus, the use of patient-derived iPS-CM models avoids potential misinterpretations arising from poorly controlled transcription levels.

One of the main disadvantages that iPS-CM present, as mentioned earlier, is their immature phenotype, as exemplified by their characteristic spontaneous beating. Their membrane potential is depolarized compared with adult cardiomyocytes, and their ultrastructural organization is poor regarding sarcomere and t-tubule development. Accordingly, they exhibit a gene expression profile similar to fetal cardiomyocyte [84]. 

Important progress in overcoming this limitation has been recently achieved. Ronaldson-Bouchard et al. [85] demonstrated that formation of tissues from early-stage iPS-CMs followed by electromechanical stimulation yielded iPS-CM with adult-like gene expression profile and organized ultrastructure, together with the development of t-tubules and functional calcium handling. These cells also presented an action potential shape with a characteristic notch, a resting membrane potential around -70 mV and, importantly, the presence of the IK1 current.

Another advantage of the iPS cell-based model system is that it offers the most accurate and relevant setting for drug toxicity screening of all the model systems, which could help exclude potential drugs, based on their toxic effects on cardiomyocytes before they go into expensive clinical trials. Lastly, since iPS cell lines are immortal and do continually express the mutant channel (unlike expression systems), they can be shared between groups, allowing for new collaborations and more in-depth characterization.

However, the generation of iPS cells requires ethical approval. Although it is possible to obtain beating clusters or sheets composed of ventricular, atrial and pacemaker cell [86], they are not organized in layers of endocardium and epicardium, and it is difficult to purify populations of ventricular or atrial cells. However, methods have been developed to direct differentiation towards a specific cardiac subtype by manipulating retinoid signal [87]. Identification of the cell surface marker VCAM1 [88] also made it possible to purify and reassemble iPS derived cardiac cells in cardiac tissue sheets with a possible application in cell therapy. 

Finally, because iPS-CM do not come from a heart, it would be arguable to attribute to them chamber-specific characteristics such as RV or LV, or layer-specific characteristics (epicardium or endocardium). This limits the interpretation of the data obtained from this model since Brugada syndrome is a chamber (RV)-specific disease. 

## 7. Conclusions

Brugada syndrome is a heterogeneous condition with an unpredictable clinical course. Several experimental approaches have been used to define the underlying mechanisms of this life-threatening disease. The major findings, advantages, and disadvantages of each model included in this review are summarized in Table 1.

The role of a defective sodium current as an underlying pathophysiological mechanism is apparent in all the models used. Many *SCN5A* mutations have been studied in heterologous expression systems, and the observed sodium current loss of function greatly agreed with the clinical findings. However, incomplete penetrance is perhaps the main interrogate remaining. 

Whole animal models coincide in that the associated arrhythmias originate in the RVOT. Furthermore, increasing evidence shows that microstructural abnormalities occur in this area of the heart, which may lead to conduction defects. These abnormalities cannot be observed in the canine wedge preparation model, in which the repolarization hypothesis is based. Thus, although differences in epicardial and endocardial AP may contribute to the development of the arrhythmia, it is probable that these differences do not explain the pathophysiology entirely. It is important to remark that in the canine wedge model pharmacological tools are needed to induce arrhythmias.

One of the unsolved questions about BrS is the lack of known genetic mutations in approximately 70% of the diagnosed patients. Numerous efforts have been made to find genes, other than *SCN5A*, implicated in the disease. Despite experimental evidence supporting the role of more than 20 genes in BrS, their role as the monogenetic cause of this disease is being disputed [89]. Moreover, increasing evidence suggests that BrS is an oligogenic disease, in which a combination of several genetic factors with different degrees of effect sizes underlie the clinical phenotype [90].

The development of iPS-CM as a model to study BrS brought the opportunity of delving into detailed cell physiology of cardiomyocytes derived from actual mutation carriers and/or diagnosed patients without an identified genetic cause. Several laboratories, including ours, have shown evidence for the loss of function of the sodium channel in carriers of mutations in *SCN5A* [77,78,82,83]. For instance, it was expected that cells from BrS patients showed abnormalities in sodium current properties, even if the patient did not have a mutation in *SCN5A* (e.g., mutations in non-coding regions leading to low expression of the protein). However, this was not observed in two different studies using iPS-CM derived from this type of patients [79,80]. This could imply that the clinical manifestation of the disease may occur in the absence of any dysfunction of the sodium current. In other words, this may suggest that, at least in some cases, BrS may no longer be considered a channelopathy. Whether this holds true for the large majority of BrS cases (i.e., those with no genetic defects) remains to be determined. In that sense, we believe iPS-CM studies will be critical in redefining the etiology of BrS. Still, these findings do not rule out that a transient physiological impairment of the sodium current may trigger arrhythmia in a susceptible individual. As more studies on iPS-CM appear, we will be able to better understand the pathophysiology of BrS at the cellular level. We expect that iPS-CM will help answer unresolved interrogates like whether incomplete penetrance is caused by protein interactions particular to each individual genetic background. Furthermore, a larger number of studies on iPS-CM from BrS patients with the negative genetic test may reveal abnormalities in their cellular phenotype. 

It is clear that the whole animal and/or whole organ studies are needed to fully understand the occurrence of arrhythmias. iPS-CM are likely to help in re-thinking animal models that better recapitulate the diversity of phenotypical patterns.

In summary, defining the cellular mechanisms that lead to the disease will help us understand differences in clinical outcomes between patients and the role of modifier genes. However, with more than 400 genetic alterations associated with Brugada syndrome, this poses an enormous challenge. Complimentary in vitro and in vivo models have added to our understanding, but a validation of these models using patient-specific iPS-CM will help reveal the true consequences of different mutations for individual patients. Functional characterization of cardiomyocytes from iPS cells bearing specific mutations will provide novel insight into the mechanisms and pathophysiology of this condition and may offer opportunities to improve the diagnosis and treatment of patients with Brugada syndrome. 

## Figures and Tables

**Table 1:** Summary of the main findings, advantages, and disadvantages of the experimental models used for studying Brugada syndrome.

Model	Major Findings	Advantages	Disadvantages
**Murine**	***SCN5A*** **^+/−^** **mouse:** ~50% reduction in I_Na_, slowed conduction, conduction block, re-entrant arrhythmias and ventricular tachycardia [22]. Age and sex-related factors in disease progression [24,25,26,27,28]. Reduced Na_v_1.5 protein expression [33,34]. Fibrosis-related to decreased Na_v_1.5 expression [34]. ***Scn5a*** **1798insD mouse:** Bradycardia, QT prolongation and right ventricular conduction slowing [39]. Strain dependence of conduction defect caused by the *Scn5a*1798insD/+ [41].	Allows integral studies from the whole animal, organ, tissue, and single cells. Ion channels can be knock out or modified.	Size and electrophysiological differences with the human heart. Profile of ion channel expression different to the human heart.
**Canine**	Correlation between epicardial notch AP amplitude and J wave amplitude [43]. Transmural dispersion of repolarization leading to ST-segment elevation [16,43]. Repolarization abnormalities associated with ST-segment elevation are located in the right ventricular outflow tract [49]. Male/female differences in susceptibility to Brugada syndrome are related to gender differences in Ito [53]. Focused application of RFA to the epicardium might be more efficient in eradicating ventricular tachycardia in Brugada syndrome patients [56].	Allows investigation of cells in epicardium and endocardium preserving their structural organization in the heart. Electrophysiological similarities with the human heart. Profile of ion channel expression similar to the human heart.	Brugada Syndrome phenotype has to be pharmacologically induced.
**Porcine**	Conduction slowing and increased susceptibility to ventricular arrhythmias [91] Lack of a clear Brugada Syndrome [91].	Allows investigation of cells in epicardium and endocardium preserving their structural organization in the heart. Electrophysiological similarities with the human heart.	Expensive. Long reproductive cycles.
**Heterologous expression**	See Supplementary Table	Relatively easy to perform. Allows detailed studies of the intrinsic biophysical properties of ion channels affected by mutations.	Lack of many specific cardiac proteins. Absence of patient-specific genetic background. Results may vary according to the cell model (e.g.,: HEK cells vs. Xenopus oocytes).
**iPS-CM**	Reduced sodium current, increased triggered AP activity, and abnormal Ca^2+^ transients. Genome editing reversed the effects of the mutation, indicating causality [78]. Patients with no mutation identified do not present current abnormalities [79,80]. Decrease in current correlated with a reduction of the maximum upstroke velocity and AP amplitude [82]. Mutation-induced changes, other than current density may appear in patient-specific iPS-CM, but not in heterologous expression recordings [83]. No changes in sodium current were observed in iPS-CM from patients without *SCN5A* mutation [79].	Patient-specific iPS-CM carry the patient’s exact genetic background. iPS-CM expression profile closely resembles that of cardiomyocytes. iPS cells are suitable for genome editing.	Immature phenotype. Depolarized membrane potential compared with adult cardiomyocytes. Negligible levels of I_K1_. Poor ultrastructural organization regarding sarcomere and t-tubule development.

## Supplementary Materials

Supplementary materials can be found at https://www.mdpi.com/1422-0067/20/9/2123/s1.

## Abbreviations

AV node	atrial ventricular node
HEK	human embryonic kidney
iPS cells	induced pluripotent stem cells
iPS-CM	induced pluripotent stem cell-derived cardiomyocytes
LQT syndrome	long QT syndrome
P2R	phase 2 reentry
I_Na_	sodium inward current
tsA201 cells	immortalized HEK293 cells
