# Supplementary material for: Experimental Models of Brugada syndrome
Source: Int J Mol Sci. 2019 Apr 29;20(9):2123. doi: 10.3390/ijms20092123 (PMC6539778; doi:10.3390/ijms20092123)
Supplement: Supplementary file 1 [file ijms-20-02123-s001.pdf]

Supplemental Table. Brugada Syndrome- associated *SCN5A* mutant channels and their reported electrophysiological properties.

Genetic variation	Expression system	Current	Activation	Inactivation	Recovery from inactivation	Other features	Reference
p.R18Q	HEK293 cells<br>Xenopus oocytes	~	~	~	~		Gütter (2013) [92]
p.R27H	HEK293 cells	~	+ shift	~	~		Gütter (2013) [92]
p.R27H	Xenopus oocytes	~	~	~	~		Gütter (2013) [92]
p.G35S	HEK293 cells<br>Xenopus oocytes	~	~	~	~		Gütter (2013) [92]
p.Q55X	tsA201 cells	No	NS	NS	NS		Makita (2007)[93]
p.V95I	HEK293 cells<br>Xenopus oocytes	~	~	~	~		Gütter (2013) [92]
p.R104Q	HEK293 cells	No	NS	NS	NS		Gütter (2013) [92]
p.R104Q	Xenopus oocytes	↓	~	- shift	↓		Gütter (2013) [92]
p.R104W	HEK293 cells<br>Rat neonatal cardiomyocytes	No	NS	NS	NS	Channel retention in the ER.	Clatot (2012) [94]
p.R121W	HEK293 cells	No	NS	NS	NS	Channel retention in the ER.	Clatot (2012)[94]

Genetic variation	Expression system	Current	Activation	Inactivation	Recovery from inactivation	Other features	Reference
	Rat neonatal cardiomyocytes						
p.A124D	tsA201 cells	↓	~	~	NS	Channel retention in the ER.	Moreau (2012)[95]
p.K126E	HEK293 cells	~	+ shift	+ shift	~		[92]
p.K126E	Xenopus oocytes	~	~	~	~		[92]
p.W156X	Xenopus oocytes	No	-	-	-		Kapplinger *(2010)*[96]; Bezzina (2003)[97]
p.E161K	tsA201 cells	↓	+ shift	~	NS		Smits (2005)[98]
	HEK293 cells Xenopus oocytes	↓	+ shift	~	~	Trafficking defect.	Gui (2010)[99]
p.R179X	HEK293 cells	No	NS	NS	NS		Kawamura (2009)[100]
p.T187I	HEK293 cells	No	NS	NS	NS		Makiyama (2005) [101]
	HEK293 cells	No	NS	NS	NS	Trafficking defect.	Gui (2010) [99]
	Xenopus oocytes	No	NS	NS	NS		
p.S216L	tsA201 cells	↓	~	~	~		Marangoni (2011) [102]
p.T220I	HEK293 cells	~	~	- shift	~	Smaller window current.	*Kapplinger (2010)*[96]*;* Beyder (2014)[103]
	HEK293 cells	↓	~	- shift	~	Bigger fast recovery time constant.	Gui (2010) [99]

Genetic variation	Expression system	Current	Activation	Inactivation	Recovery from inactivation	Other features	Reference
						Correct trafficking.	
	Xenopus oocytes	↓	~	- shift	~		
p.R222X	Xenopus oocytes	↓	~	~	~		Ortiz-Bonnin (2016)[104]
p.R225W	Xenopus oocytes	↓	+ shift	+ shift	-		Kapplinger (2010)[96]; Bezzina (2003)[97]
p.A226V	tsA201 cells	↓	NS	NS	NS		Tan (2015)[105]
p.Q270K	CHO-K1 cells	↓	+ shift	+ shift	~	Slower decay current.	Calloe (2011)[106]
p.R282H	HEK293 cells	↓	+ shift	+ shift	~	Reduced single channel conductance. Trafficking defect.	Itoh (2005a)[107]
p.R282H	HEK293 cells	No	NS	NS	NS		Shinlapawittayatorn (2011)[108]
p.G292S	HEK293 cells	↓	NS	+ shift	~		Niimura (2004)[109]; Shinlapawittayatorn (2011)[108]
	HEK293 cells	↓	NS	NS	NS		Zhang (2015)[110]
p.V294M	HEK293 cells	↓	NS	~	~		Priori (2002)[111]; Shinlapawittayatorn (2011)[108]
	HEK293 cells	↓	NS	NS	NS		Zhang (2015)[110]
p.K317N	HEK293 cells	No	NS	NS	NS		*Yi (2003)[112]* Shinlapawittayatorn (2011[108])
p.K317N	HEK293 cells	↓	NS	NS	NS		Zhang (2015)[110]

Genetic variation	Expression system	Cur-rent	Activa-tion	Inactiva-tion	Recovery from inac-tivation	Other features	Reference
p.L325R	HEK293 cells	↓	+ shift	+ shift	NS	Slower time to peak and onset of fast inactivation.	Keller (2005)[66]
p.L325R	HEK293 cells	No	NS	NS	NS		Shinlapawittayatorn (2011)[108]<br>Clatot (2018)[113]
p.P336L	tsA201 cells	↓	~	~	NS		Cordeiro (2006)[114]
p.G351V	Xenopus oocytes	No	NS	NS	NS		Vatta (2002)[67]
p.G351V	tsA201 cells	↓	~	~	~		Vatta (2002)[67]
p.G351V	HEK293 cells	No	NS	NS	NS		Shinlapawittayatorn (2011[108])
p.T353I	HEK293 cells	↓	~	- shift	NS	Trafficking defect.	Pfahnl (2007)[115]
p.D356N	HEK293 cells	No	NS	NS	NS		Makiyama (2005)[101]; Shin-lapawittayatorn (2011[108])
p.R367C	HEK293 cells	No	NS	NS	NS		Smits (2002[116]);<br>Meregalli (2009)[117]
p.R367H	HEK293 cells	No	NS	NS	NS		Hong (2004),[118] Shinlapawittayatorn (2011)[108]
	Xenopus oocytes	No	NS	NS	NS		Vatta (2002)[119];<br>Takehara (2004[120])
p.R376H	HEK293 cells	↓	~	~	NS		Rossenbacker (2004)[121]
p.R376H	HEK293 cells	↓	NS	NS	NS		Frustaci (2005)[122]

Genetic variation	Expression system	Current	Activation	Inactivation	Recovery from inactivation	Other features	Reference
p.R376H	HEK293 cells	No	NS	NS	NS		Shinlapawittayatorn (2011)[108]
p.N406S	HEK293 cells	~	+ shift	+ shift	↓	Larger time constant for the slow recovery component, but faster recovery from fast inactivation. Normal trafficking.	Itoh (2005b)[123]
p.E473X	tsA201 cells	No	NS	NS	NS		Baroudi (2004)[59]
p.R526H	HEK293 cells	~	~	~	~	Normal trafficking.	Aiba (2014)[124]
p.R535X	HEK293 cells	No	NS	NS	NS		Keller (2005a)[64]
p.A551T	tsA201 cells	↓	~	- shift	~		Chiang (2009)[125]
	HEK293 cells	↓	- shift	~	NS	Smaller activation slope.	Juang (2014)[126]
p.L567Q	HEK293 cells	↓	+ shift	- shift	~		Wan (2001)[65]
p.N592K	HEK293 cells	↓	+ shift	~	NS		Juang (2014)[126]
p.G615E	HEK293 cells	~	+ shift	~	~	Smaller inactivation slope.	Kapplinger (2010)[96]; Beyder (2014)[103]
	Xenopus oocytes	~	~	- shift	↑		Albert (2008)[127]
p.R620H	CHO-K1 cells	~	~	~	~		Calloe (2013)[128]
p.P648L	HEK293 cells	~	~	~	~	Smaller time constant of slow inactivation.	Kapplinger (2010)[96]; Beyder (2014)[103]
p.H681P	tsA201 cells	↓	- shift	- shift	~	Reduced window current.	Mok (2003)[129]

Genetic variation	Expression system	Current	Activation	Inactivation	Recovery from inactivation	Other features	Reference
p.A735V	Xenopus oocytes	~	+ shift	~	↓		Vatta (2002)[119]
p.G752R	COS-7 cells	↓	+ shift	+ shift	NS		Potet (2003)[130]
p.R811H	CHO-K1 cells	↓	~	- shift	↓		Calloe (2013)[128]
p.L812Q	HEK293 cells	↓	~	- shift	~	Smaller inactivation slope. Intracellular retention.	Wang (2015)[131]
p.R814Q	Xenopus oocytes	NA	~	- shift	NS	Smaller activation and inactivation slopes.	Frigo (2007)[132]; Chen (1996)[133]
p.K817E	HEK293 cells	↓	+ shift	~	↓	Bigger activation and inac-tivation slopes. Good trafficking. Similar single-channel conductance.	Kinoshita (2016)[134]
p.W822X	tsA201 cells	No	NS	NS	NS	No current.	Keller (2005b)[135]
	HEK293 cells	No	NS	NS	NS	Trafficking defect.	Teng (2009)[136]
p.I848fs	tsA201 cells	↓	NS	NS	NS		Hsueh (2009)[137]
p.R878C	Xenopus oocytes	↓	~	~	~		Zhang (2008)[51][138]
p.R878C	HEK293 cells	No	NS	NS	NS	Normal trafficking.	Zhang (2008)[51][138]
p.R878C	Xenopus oocytes	No	NS	NS	NS		Gui (2010[99])
p.R878C	HEK293 cells	No	NS	NS	NS	Normal trafficking.	
p.I890T	HEK293 cells	↓	+ shift	~	~	Normal trafficking.	Tarradas (2013)[63]

Genetic variation	Expression system	Cur-rent	Activa-tion	Inactiva-tion	Recovery from inac-tivation	Other features	Reference
p.R965C	tsA201 cells	~	~	- shift	↓		Hsueh (2009)[137]
p.A997T	HEK293 cells	↓	+ shift	+ shift	~	Bigger activation slope, smaller in-activation slope, smaller window current.	Kapplinger (2010)[139]; Beyder (2014)[103]
p.R1023H	HEK293 cells	↓	~	~	~		Frustaci (2005)[122]
p.E1053K	HEK293 cells	~	- shift	- shift	↓	Faster onset of in-activation.	Mohler (2004)[140]
p.W1191X	tsA201 cells	No	NS	NS	NS		Shin (2007[60])
p.R1193Q	Xenopus oocytes	~	~	+ shift	~		Vatta (2002)[119]
	tsA201 cells	~	~	- shift	NS		Huang (2006)[141]
	Xenopus oocytes	~	~	- shift			Wang (2004)[142]
p.S1218I	CHO-K1 cells	No	NS	NS	NS		Calloe (2013)[128]
p.D1275N	HEK293 cells	↓	NS	NS	NS		Kapplinger (2010)[96]; Zhang (2015)[110]
	HEK293 cells	↓	+ shift	~	↑	Bigger inactiva-tion slope, de-creased fast re-covery time con-stant. Trafficking defect.	Gui (2010)[99]
	Xenopus oocytes	↓	+ shift	- shift	↑	Bigger activation and inactivation slope, smaller fast recovery time constant.	

Genetic variation	Expression system	Current	Activation	Inactivation	Recovery from inactivation	Other features	Reference
	Xenopus oocytes	~	+ shift	~	↑		Groenewegen (2002)[143]
p.G1319V	HEK293 cells	~	+ shift	- shift	↓		Casini (2007)[144]
p.V1328M	HEK293 cells	~	~	+ shift	~		Turker (2016)[145]
p.V1340I	HEK293 cells	No	NS	NS	NS	Normal trafficking.	Samani (2009)[146]
p.F1344S	tsA201 cells	~	+ shift	~	↑		Keller (2006)[135]
p.V1378M	tsA201 cells	↓	~	~	NS	Channel retention in the ER.	Moreau (2012)[95]
p.L1393X	NIH-3T3 cells HEK293 cells	No	NS	NS	NS	Normal trafficking.	Samani (2009)[147]
p.K1397delA	Xenopus oocytes	No	NS	NS	NS		Chen (1998)[2]
p.G1406R	COS-7cells	No	NS	NS	NS	Normal trafficking.	Kyndt (2001)[148]
p.G1406R	HEK293 cells	↓	NS	NS	NS	Trafficking defect.	Kapplinger *(2010)*[96]; Tan (2006)[149]
p.G1408R	HEK293 cells Xenopus oocytes	No	NS	NS	NS		Kapplinger (2010)[96]; Gui (2010)[99]
p.A1427S	HEK293 cells	↓	+ shift	~	NS		Xiong (2014)[150]
p.A1428S	HEK293 cells	↓	~	~	~		Zhu (2015)[151]
p.D1430N	tsA201 cells	No	NS	NS	NS	Normal trafficking.	Maury (2013) [152]

Genetic variation	Expression system	Cur-rent	Activa-tion	Inactiva-tion	Recovery from inac-tivation	Other features	Reference
p.R1432G	tsA201 cells	No	NS	NS	NS		Deschênes (2000)[153]
p.R1432G	tsA201 cells	No	NS	NS	NS	Channel retention in the ER.	Baroudi (2001) [58]
p.R1432G	Xenopus oocytes	~	~	NS	NS		Baroudi (2001) [58]
p.P1438L	tsA201 cells	No		NS	NS		Six (2008)[154]
p.Q1476X	tsA201 cells	No	NS	NS	NS		Maury (2013) [152])
p.1493delK	HEK293 cells	↓	~	~	↑	Bigger activation slope and smaller inactivation slope, decreased fast re-covery time con-stant.<br>Channel retention in the ER.	Kapplinger (2010)[96];<br>Zumhagen (2014)[155]
	HEK293-EBNA cells	↓	+ shift	+ shift	↑	Bigger activation slope.	Zhang (2007)[156]
p.1500delK	HEK293-EBNA cells	~	+ shift	- shift	NS	Bigger activation and inactivation slopes.	Priori (2002)[111];<br>Grant (2002)[157]
p.1505S	HEK293 cells	↓	+ shift	- shift	NS	Bigger activation slope and smaller inactivation slope	Saber (2015)[158]
delKPQ 1505-1507	Xenopus oocytes	~	NA	- shift		Decreased cur-rent decay.	Postema (2011)[159];<br>Bennett (1995)[160]
	tsA201 cells	~	+ shift	~	↑	Bigger activation slope.	Wang (1996)[161]
	HEK293-EBNA cells	↑	~	~	↑		Chandra (1998)[162]
	HEK293 cells	~	+ shift	~	↑		Nagatomo (1998)[163]

Genetic variation	Expression system	Current	Activation	Inactivation	Recovery from inactivation	Other features	Reference
	tsA201 cells	NA	+ shift	- shift			Makita (2008a)[164]
	Cardiomyocytes derived from murine iPSc	~	~	~	↑		Malan (2011)[165]
p.R1512W	tsA201 cells	~	~	~	↓	Slower current decay.	Deschênes (2000)[153]
	Xenopus oocytes	NA	- shift	- shift	~		Rook (1999)[68]
	HEK293 cells	↓	~	~	~		Zheng (2016)[166]
p.K1527R/ p.A1569P	tsA201 cells	~	~	- shift	~		Yokoi (2005)[167]
p.K1578dupAA	HEK293 cells	No	NS	NS	NS		Makiyama (2005)
	HEK293 cells	No	NS	NS	NS		Gui (2010) [99]
p.1617delF	HEK293 cells	~	- shift	- shift	↑	Faster current decay, bigger activation slope. Normal trafficking	Liang (2006)[168]; Gui (2010)[99]
	tsA201 cells	~	~	- shift	NS	Faster decay at <-40mV.	Chen (2005)[169]
p.T1620M	tsA201 cells	~	+ shift	~	↓	Positive shift in activation (at 32ºC, not at 22ºC).	Dumaine (1999)[170]
	tsA201 cells	~	~	+ shift	↓	Enhanced development of intermediate inactivation.	Wang (2000)[171]
	tsA201 cells	~	~	~	↓		Baroudi (2000a)[172]

Genetic variation	Expression system	Current	Activation	Inactivation	Recovery from inactivation	Other features	Reference
	Xenopus oocytes	~	~	+ shift	↑		
	Xenopus oocytes	~	~	+ shift	~		Makita (2000)[70]
	tsA201 cells	~	+ shift	+ shift	~		Shirai (2002)[173]
p.R1232W/ p.T1620M	Xenopus oocytes	NA	~	+ shift	↑		Chen (1998)[2]
p.R1232W/ p.T1620M	Xenopus oocytes	~	~	+ shift	↑		Vilin (2001)[174]
p.R1232W/ p.T1620M	tsA201 cells	No	NS	NS	NS	Channel retention in the ER.	Baroudi (2002)[175]
p.R1232W/ p.T1620M	tsA201 cells	~	~	+ shift	NS	Normal trafficking.	Makita (2008b)[176]
p.R1623X	HEK293 cells	No	NS	NS	NS		Makiyama (2005)[101]
	HEK293 cells	No	NS	NS	NS	Trafficking defect.	Gui (2010) [99]
p.R1629Q	HEK293 cells	~	~	- shift	↓	Faster onset of inactivation.	Zeng (2013)[177]
p.R1629X	tsA201 cells	↓	NS	NS	NS		Tan (2015)[105]
p.R1632C	tsA201 cells	↓	~	- shift	↓		Nakajima (2015)[178]
p.R1644C	HEK293 cells	~	+ shift	~	↑		Frustaci (2005)[122]
p.A1649V	tsA201 cells	NA	- shift	- shift	↓		Liang (2006)[168]; Tang (1998)[179]
p.I1660V	tsA201 cells	↓	NS	NS	NS	Trafficking defect.	Cordeiro (2006)[114]
p.A1680del-TTA insC	HEK293 cells	No	NS	NS	NS		Kranjcec (2007)[180]
p.D1690N	CHO cells	↓	~	~	~	Trafficking defect.	Núnez (2013[181])

Genetic variation	Expression system	Cur-rent	Activa-tion	Inactiva-tion	Recovery from inac-tivation	Other features	Reference
	HEK293 cells	↓	+ shift	~	↓		Zeng (2016)[182]
p.S1710L	tsA201 cells	~	+ shift	- shift	↓		Shirai (2002)[173]
p.G1712C	HEK293 cells	No	NS	NS	NS		Chen (2016)[183]
p.D1714G	HEK293 cells	↓	~	~	~		Amin (2005)[184]
p.G1740R	tsA201 cells	No	NS	NS	NS	Trafficking defect.	Baroudi (2004)[59]
p.G1743E	tsA201 cells	↓	NS	NS	NS		Vernooy (2006)[185]
p.G1743R	HEK293 cells	No	NS	NS	NS	Trafficking defect.	Valdivia (2004) [186]
p.G1743R	HEK293 cells	No	NS	NS	NS		Zhang (2015)[110]
p.G1748D	CHO cells	↓	+ shift	+ shift	↑	Trafficking defect.	Núnez (2013)[181]
p.N1774delT	tsA201 cells	No	NS	NS	NS		Baroudi (2004)[59]
p.E1784K	Xenopus oocytes	~	NS	- shift	~		Nakajima (2011)[187];<br>Wei (1999)[188]
	tsA201 cells	↓	+ shift	- shift	~	Bigger activation slope, faster cur-rent decay.<br>Correct traffick-ing.	Makita (2008a)[164]
	tsA201 cells	~	+ shift	- shift	↑		Deschênes (2000)[153]
	tsA201 cells	↓	~	- shift	NS		Veltmann (2016)[189]
p.L1786Q	HEK293 cells	↓	+ shift	- shift	NS	Bigger activation slope.	Hofman-Bang (2006)[190];<br>Kanters (2014)[191]

Genetic variation	Expression system	Current	Activation	Inactivation	Recovery from inactivation	Other features	Reference
p.1795insD	Xenopus oocytes	↓	+ shift	- shift	↓		Bezzina (1999)[62]
	tsA201 cells	↓	+ shift	- shift	NS		Baroudi (2000b)[61]
	HEK293 cells	~	~	- shift	~		Veldkamp (2000)[69]
p.Y1795H	HEK293 cells	↓	~	- shift	~		Rivolta (2001)[192]
p.1816insT	CHO cells	↓	+ shift	+ shift	↑	Decreased time to peak, decreased window current. Trafficking defect.	Dolz-Gaitón (2014)[193]
p.Q1832E	HEK293 cells	↓	~	~	~	Trafficking defect.	Gando (2017)[194]
p.C1850S	HEK293 cells	↓	~	- shift	~	Accelerated fast inactivation.	Petitprez (2008)[195]
p.1876dupATG	tsA201 cells	~	+ shift	- shift	↓	Bigger activation slope. Bigger inactivation time constants.	Hsueh (2009)[137]
p.A1924T	Xenopus oocytes	NA	- shift	~	~		Rook (1999)[68]
p.G1935S	HEK293 cells	~	~	~	~		Coronel (2005)[3]
p.V1951L	tsA201 cells	↑	~	~	↑		Priori (2002)[111]; Wang (2007)[196]
p.I1968S	HEK293 cells	↓	~	- shift	↓	Slower onset of inactivation.	Frustaci (2005)[122]
p.F2004L	CHO cells	↓	~	- shift	↓	Slower time constant of the fast component of recovery from inactivation.	Bébarová (2008)[197]
	tsA201 cells	~	~	- shift	↑		Wang (2007)[196]

Genetic variation	Expression system	Cur-rent	Activa-tion	Inactiva-tion	Recovery from inac-tivation	Other features	Reference
p.R2012H	Xenopus oocytes	↓	~	- shift	~		Ortiz-Bonnin (2016)[104]

Table showing the available data for the electrophysiological studies published for mutations in *SCN5A* that have been associated with Brugada Syndrome. Databases searched are Biobase-Human Gene Mutation Database (http://www.biobase-international.com/product/hgmd), The gene connection for the heart (http://www. fsm.it/cardmoc/), and PubMed (http://www.ncbi.nlm.nih.gov/pubmed). HEK293 cells, Human Embryonic Kidney cells; tsA201 cells, immortalized HEK293 cells; HEK293-EBNA, transformed HEK293 cells; CHO cells, Chinese Hamster Ovary cells; COS-7cells, African green monkey kidney fibroblast-like cell line; iPSc, induced Pluripotent Stem cells; ~, no change; ↓ reduced/decelerated; ↑ increased/accelerated; NS not studied; NA not available; ER endoplasmic reticulum. Activation and inactivation data refer to $V_{1/2}$. References in italics correspond to the association of the given mutation with the disease and are provided in case the report of the functional characterization does not specify the disease or refers to other diseases. Dark gray highlights data of mutations for which a complete loss of current was reported, and mutations with described changes in the electrophysiological properties of the channel are marked in light gray

References

92. Gutter, C.; Benndorf, K.; Zimmer, T. Characterization of N-terminally mutated cardiac Na(+) channels associated with long QT syndrome 3 and Brugada syndrome. *Front Physiol* **2013**, *4*, 153.
93. Makita, N.; Sumitomo, N.; Watanabe, I.; Tsutsui, H. Novel SCN5A mutation (Q55X) associated with age-dependent expression of Brugada syndrome presenting as neurally mediated syncope. *Heart rhythm* **2007**, *4*, 516-519.
94. Clatot, J.; Ziyadeh-Isleem, A.; Maugenre, S.; Denjoy, I.; Liu, H.; Dilanian, G.; Hatem, S.N.; Deschenes, I.; Coulombe, A.; Guicheney, P., et al. Dominant-negative effect of SCN5A N-terminal mutations through the interaction of Na(v)1.5 alpha-subunits. *Cardiovascular research* **2012**, *96*, 53-63.
95. Moreau, A.; Keller, D.I.; Huang, H.; Fressart, V.; Schmied, C.; Timour, Q.; Chahine, M. Mexiletine differentially restores the trafficking defects caused by two brugada syndrome mutations. *Front Pharmacol* **2012**, *3*, 62.
96. Kapplinger, J.D.; Tester, D.J.; Alders, M.; Benito, B.; Berthet, M.; Brugada, J.; Brugada, P.; Fressart, V.; Guerchicoff, A.; Harris-Kerr, C., et al. An international compendium of mutations in the SCN5A-encoded cardiac sodium channel in patients referred for Brugada syndrome genetic testing. *Heart rhythm* **2010**, *7*, 33-46.
97. Bezzina, C.R.; Rook, M.B.; Groenewegen, W.A.; Herfst, L.J.; van der Wal, A.C.; Lam, J.; Jongsma, H.J.; Wilde, A.A.; Mannens, M.M. Compound heterozygosity for mutations (W156X and R225W) in SCN5A associated with severe cardiac conduction disturbances and degenerative changes in the conduction system. *Circ Res* **2003**, *92*, 159-168.
98. Smits, J.P.; Koopmann, T.T.; Wilders, R.; Veldkamp, M.W.; Opthof, T.; Bhuiyan, Z.A.; Mannens, M.M.; Balser, J.R.; Tan, H.L.; Bezzina, C.R., et al. A mutation in the human cardiac sodium channel (E161K) contributes to sick sinus syndrome, conduction disease and Brugada syndrome in two families. *Journal of molecular and cellular cardiology* **2005**, *38*, 969-981.
99. Gui, J.; Wang, T.; Jones, R.P.; Trump, D.; Zimmer, T.; Lei, M. Multiple loss-of-function mechanisms contribute to SCN5A-related familial sick sinus syndrome. *PloS one* **2010**, *5*, e10985.

100. Kawamura, M.; Ozawa, T.; Yao, T.; Ashihara, T.; Sugimoto, Y.; Yagi, T.; Itoh, H.; Ito, M.; Makiyama, T.; Horie, M. Dynamic change in ST-segment and spontaneous occurrence of ventricular fibrillation in Brugada syndrome with a novel nonsense mutation in the SCN5A gene during long-term follow-up. *Circ J* **2009**, *73*, 584-588.
101. Makiyama, T.; Akao, M.; Tsuji, K.; Doi, T.; Ohno, S.; Takenaka, K.; Kobori, A.; Ninomiya, T.; Yoshida, H.; Takano, M., et al. High risk for bradyarrhythmic complications in patients with Brugada syndrome caused by SCN5A gene mutations. *Journal of the American College of Cardiology* **2005**, *46*, 2100-2106.
102. Marangoni, S.; Di Resta, C.; Rocchetti, M.; Barile, L.; Rizzetto, R.; Summa, A.; Severi, S.; Sommariva, E.; Pappone, C.; Ferrari, M., et al. A Brugada syndrome mutation (p.S216L) and its modulation by p.H558R polymorphism: standard and dynamic characterization. *Cardiovascular research* **2011**, *91*, 606-616.
103. Beyder, A.; Mazzone, A.; Strege, P.R.; Tester, D.J.; Saito, Y.A.; Bernard, C.E.; Enders, F.T.; Ek, W.E.; Schmidt, P.T.; Dlugosz, A., et al. Loss-of-function of the voltage-gated sodium channel NaV1.5 (channelopathies) in patients with irritable bowel syndrome. *Gastroenterology* **2014**, *146*, 1659-1668.
104. Ortiz-Bonnin, B.; Rinne, S.; Moss, R.; Streit, A.K.; Scharf, M.; Richter, K.; Stober, A.; Pfeufer, A.; Seemann, G.; Kaab, S., et al. Electrophysiological characterization of a large set of novel variants in the SCN5A-gene: identification of novel LQTS3 and BrS mutations. *Pflugers Archiv : European journal of physiology* **2016**, *468*, 1375-1387.
105. Tan, B.Y.; Yong, R.Y.; Barajas-Martinez, H.; Dumaine, R.; Chew, Y.X.; Wasan, P.S.; Ching, C.K.; Ho, K.L.; Gan, L.S.; Morin, N., et al. A Brugada syndrome proband with compound heterozygote SCN5A mutations identified from a Chinese family in Singapore. *Europace : European pacing, arrhythmias, and cardiac electrophysiology : journal of the working groups on cardiac pacing, arrhythmias, and cardiac cellular electrophysiology of the European Society of Cardiology* **2016**, *18*, 897-904.
106. Calloe, K.; Schmitt, N.; Grubb, S.; Pfeiffer, R.; David, J.P.; Kanter, R.; Cordeiro, J.M.; Antzelevitch, C. Multiple arrhythmic syndromes in a newborn, owing to a novel mutation in SCN5A. *Can J Physiol Pharmacol* **2011**, *89*, 723-736.
107. Itoh, H.; Shimizu, M.; Mabuchi, H.; Imoto, K. Clinical and electrophysiological characteristics of Brugada syndrome caused by a missense mutation in the S5-pore site of SCN5A. *Journal of cardiovascular electrophysiology* **2005**, *16*, 378-383.
108. Shinlapawittayatorn, K.; Dudash, L.A.; Du, X.X.; Heller, L.; Poelzing, S.; Ficker, E.; Deschenes, I. A novel strategy using cardiac sodium channel polymorphic fragments to rescue trafficking-deficient SCN5A mutations. *Circ Cardiovasc Genet* **2011**, *4*, 500-509.
109. Niimura, H.; Matsunaga, A.; Kumagai, K.; Ohwaki, K.; Ogawa, M.; Noguchi, H.; Yonemura, K.; Saku, K. Genetic analysis of Brugada syndrome in Western Japan: two novel mutations. *Circ J* **2004**, *68*, 740-746.
110. Zhang, J.; Chen, Y.; Yang, J.; Xu, B.; Wen, Y.; Xiang, G.; Wei, G.; Zhu, C.; Xing, Y.; Li, Y. Electrophysiological and trafficking defects of the SCN5A T353I mutation in Brugada syndrome are rescued by alpha-allocryptopine. *Eur J Pharmacol* **2015**, *746*, 333-343.
111. Priori, S.G.; Napolitano, C.; Gasparini, M.; Pappone, C.; Della Bella, P.; Giordano, U.; Bloise, R.; Giustetto, C.; De Nardis, R.; Grillo, M., et al. Natural history of Brugada syndrome: insights for risk stratification and management. *Circulation* **2002**, *105*, 1342-1347.
112. Yi, S.D.; Meng, S.R.; Cui, Y.K.; Chen, Z.M.; Peng, J. [PCR-based site-directed mutagenesis and recombinant expression plasmid construction of a SCN5A mutation (K317N) identified in a Chinese family with Brugada syndrome]. *Di Yi Jun Yi Da Xue Xue Bao* **2003**, *23*, 1139-1142.
113. Clatot, J.; Zheng, Y.; Girardeau, A.; Liu, H.; Laurita, K.R.; Marionneau, C.; Deschenes, I. Mutant voltage-gated Na(+) channels can exert a dominant negative effect through coupled gating. *Am J Physiol Heart Circ Physiol* **2018**, *315*, H1250-H1257.
114. Cordeiro, J.M.; Barajas-Martinez, H.; Hong, K.; Burashnikov, E.; Pfeiffer, R.; Orsino, A.M.; Wu, Y.S.; Hu, D.; Brugada, J.; Brugada, P., et al. Compound heterozygous mutations P336L and I1660V in the human cardiac sodium channel associated with the Brugada syndrome. *Circulation* **2006**, *114*, 2026-2033.
115. Pfahnl, A.E.; Viswanathan, P.C.; Weiss, R.; Shang, L.L.; Sanyal, S.; Shusterman, V.; Kornblit, C.; London, B.; Dudley, S.C., Jr. A sodium channel pore mutation causing Brugada syndrome. *Heart rhythm* **2007**, *4*, 46-53.

116. Smits, J.P.; Eckardt, L.; Probst, V.; Bezzina, C.R.; Schott, J.J.; Remme, C.A.; Haverkamp, W.; Breithardt, G.; Escande, D.; Schulze-Bahr, E., et al. Genotype-phenotype relationship in Brugada syndrome: electrocardiographic features differentiate SCN5A-related patients from non-SCN5A-related patients. *Journal of the American College of Cardiology* **2002**, *40*, 350-356.
117. Meregalli, P.G.; Tan, H.L.; Probst, V.; Koopmann, T.T.; Tanck, M.W.; Bhuiyan, Z.A.; Sacher, F.; Kyndt, F.; Schott, J.J.; Albuisson, J., et al. Type of SCN5A mutation determines clinical severity and degree of conduction slowing in loss-of-function sodium channelopathies. *Heart rhythm* **2009**, *6*, 341-348.
118. Hong, K.; Berruezo-Sanchez, A.; Poungvarin, N.; Oliva, A.; Vatta, M.; Brugada, J.; Brugada, P.; Towbin, J.A.; Dumaine, R.; Pinero-Galvez, C., et al. Phenotypic characterization of a large European family with Brugada syndrome displaying a sudden unexpected death syndrome mutation in SCN5A. *Journal of cardiovascular electrophysiology* **2004**, *15*, 64-69.
119. Vatta, M.; Dumaine, R.; Varghese, G.; Richard, T.A.; Shimizu, W.; Aihara, N.; Nademanee, K.; Brugada, R.; Brugada, J.; Veerakul, G., et al. Genetic and biophysical basis of sudden unexplained nocturnal death syndrome (SUNDS), a disease allelic to Brugada syndrome. *Hum Mol Genet* **2002**, *11*, 337-345.
120. Takehara, N.; Makita, N.; Kawabe, J.; Sato, N.; Kawamura, Y.; Kitabatake, A.; Kikuchi, K. A cardiac sodium channel mutation identified in Brugada syndrome associated with atrial standstill. *J Intern Med* **2004**, *255*, 137-142.
121. Rossenbacker, T.; Carroll, S.J.; Liu, H.; Kuiperi, C.; de Ravel, T.J.; Devriendt, K.; Carmeliet, P.; Kass, R.S.; Heidbuchel, H. Novel pore mutation in SCN5A manifests as a spectrum of phenotypes ranging from atrial flutter, conduction disease, and Brugada syndrome to sudden cardiac death. *Heart rhythm* **2004**, *1*, 610-615.
122. Frustaci, A.; Priori, S.G.; Pieroni, M.; Chimenti, C.; Napolitano, C.; Rivolta, I.; Sanna, T.; Bellocci, F.; Russo, M.A. Cardiac histological substrate in patients with clinical phenotype of Brugada syndrome. *Circulation* **2005**, *112*, 3680-3687.
123. Itoh, H.; Shimizu, M.; Takata, S.; Mabuchi, H.; Imoto, K. A novel missense mutation in the SCN5A gene associated with Brugada syndrome bidirectionally affecting blocking actions of antiarrhythmic drugs. *Journal of cardiovascular electrophysiology* **2005**, *16*, 486-493.
124. Aiba, T.; Farinelli, F.; Kostecki, G.; Hesketh, G.G.; Edwards, D.; Biswas, S.; Tung, L.; Tomaselli, G.F. A mutation causing Brugada syndrome identifies a mechanism for altered autonomic and oxidant regulation of cardiac sodium currents. *Circ Cardiovasc Genet* **2014**, *7*, 249-256.
125. Chiang, K.C.; Lai, L.P.; Shieh, R.C. Characterization of a novel Nav1.5 channel mutation, A551T, associated with Brugada syndrome. *J Biomed Sci* **2009**, *16*, 76.
126. Juang, J.M.; Lu, T.P.; Lai, L.C.; Hsueh, C.H.; Liu, Y.B.; Tsai, C.T.; Lin, L.Y.; Yu, C.C.; Hwang, J.J.; Chiang, F.T., et al. Utilizing multiple in silico analyses to identify putative causal SCN5A variants in Brugada syndrome. *Scientific reports* **2014**, *4*, 3850.
127. Albert, C.M.; Nam, E.G.; Rimm, E.B.; Jin, H.W.; Hajjar, R.J.; Hunter, D.J.; MacRae, C.A.; Ellinor, P.T. Cardiac sodium channel gene variants and sudden cardiac death in women. *Circulation* **2008**, *117*, 16-23.
128. Calloe, K.; Refaat, M.M.; Grubb, S.; Wojciak, J.; Campagna, J.; Thomsen, N.M.; Nussbaum, R.L.; Scheinman, M.M.; Schmitt, N. Characterization and mechanisms of action of novel NaV1.5 channel mutations associated with Brugada syndrome. *Circulation. Arrhythmia and electrophysiology* **2013**, *6*, 177-184.
129. Mok, N.S.; Priori, S.G.; Napolitano, C.; Chan, N.Y.; Chahine, M.; Baroudi, G. A newly characterized SCN5A mutation underlying Brugada syndrome unmasked by hyperthermia. *Journal of cardiovascular electrophysiology* **2003**, *14*, 407-411.
130. Potet, F.; Mabo, P.; Le Coq, G.; Probst, V.; Schott, J.J.; Airaud, F.; Guihard, G.; Daubert, J.C.; Escande, D.; Le Marec, H. Novel brugada SCN5A mutation leading to ST segment elevation in the inferior or the right precordial leads. *Journal of cardiovascular electrophysiology* **2003**, *14*, 200-203.
131. Wang, L.; Meng, X.; Yuchi, Z.; Zhao, Z.; Xu, D.; Fedida, D.; Wang, Z.; Huang, C. De Novo Mutation in the SCN5A Gene Associated with Brugada Syndrome. *Cell Physiol Biochem* **2015**, *36*, 2250-2262.
132. Frigo, G.; Rampazzo, A.; Bauce, B.; Pilichou, K.; Beffagna, G.; Danieli, G.A.; Nava, A.; Martini, B. Homozygous SCN5A mutation in Brugada syndrome with monomorphic ventricular tachycardia and

structural heart abnormalities. *Europace : European pacing, arrhythmias, and cardiac electrophysiology : journal of the working groups on cardiac pacing, arrhythmias, and cardiac cellular electrophysiology of the European Society of Cardiology* **2007**, *9*, 391-397.
133. Chen, L.Q.; Santarelli, V.; Horn, R.; Kallen, R.G. A unique role for the S4 segment of domain 4 in the inactivation of sodium channels. *J Gen Physiol* **1996**, *108*, 549-556.
134. Kinoshita, K.; Takahashi, H.; Hata, Y.; Nishide, K.; Kato, M.; Fujita, H.; Yoshida, S.; Murai, K.; Mizumaki, K.; Nishida, K., et al. SCN5A(K817E), a novel Brugada syndrome-associated mutation that alters the activation gating of NaV1.5 channel. *Heart rhythm* **2016**, *13*, 1113-1120.
135. Keller, D.I.; Huang, H.; Zhao, J.; Frank, R.; Suarez, V.; Delacretaz, E.; Brink, M.; Osswald, S.; Schwick, N.; Chahine, M. A novel SCN5A mutation, F1344S, identified in a patient with Brugada syndrome and fever-induced ventricular fibrillation. *Cardiovascular research* **2006**, *70*, 521-529.
136. Teng, S.; Gao, L.; Paajanen, V.; Pu, J.; Fan, Z. Readthrough of nonsense mutation W822X in the SCN5A gene can effectively restore expression of cardiac Na+ channels. *Cardiovascular research* **2009**, *83*, 473-480.
137. Hsueh, C.H.; Chen, W.P.; Lin, J.L.; Tsai, C.T.; Liu, Y.B.; Juang, J.M.; Tsao, H.M.; Su, M.J.; Lai, L.P. Distinct functional defect of three novel Brugada syndrome related cardiac sodium channel mutations. *J Biomed Sci* **2009**, *16*, 23.
138. Zhang, Y.; Wang, T.; Ma, A.; Zhou, X.; Gui, J.; Wan, H.; Shi, R.; Huang, C.; Grace, A.A.; Huang, C.L., et al. Correlations between clinical and physiological consequences of the novel mutation R878C in a highly conserved pore residue in the cardiac Na+ channel. *Acta Physiol (Oxf)* **2008**, *194*, 311-323.
139. Kapplinger, J.D.; Pundi, K.N.; Larson, N.B.; Callis, T.E.; Tester, D.J.; Bikker, H.; Wilde, A.A.M.; Ackerman, M.J. Yield of the RYR2 Genetic Test in Suspected Catecholaminergic Polymorphic Ventricular Tachycardia and Implications for Test Interpretation. *Circulation. Genomic and precision medicine* **2018**, *11*, e001424.
140. Mohler, P.J.; Rivolta, I.; Napolitano, C.; LeMaillet, G.; Lambert, S.; Priori, S.G.; Bennett, V. Nav1.5 E1053K mutation causing Brugada syndrome blocks binding to ankyrin-G and expression of Nav1.5 on the surface of cardiomyocytes. *Proc Natl Acad Sci U S A* **2004**, *101*, 17533-17538.
141. Huang, H.; Zhao, J.; Barrane, F.Z.; Champagne, J.; Chahine, M. Nav1.5/R1193Q polymorphism is associated with both long QT and Brugada syndromes. *Can J Cardiol* **2006**, *22*, 309-313.
142. Wang, Q.; Chen, S.; Chen, Q.; Wan, X.; Shen, J.; Hoeltge, G.A.; Timur, A.A.; Keating, M.T.; Kirsch, G.E. The common SCN5A mutation R1193Q causes LQTS-type electrophysiological alterations of the cardiac sodium channel. *J Med Genet* **2004**, *41*, e66.
143. Groenewegen, W.A.; Firouzi, M.; Bezzina, C.R.; Vliex, S.; van Langen, I.M.; Sandkuijl, L.; Smits, J.P.; Hulsbeek, M.; Rook, M.B.; Jongsma, H.J., et al. A cardiac sodium channel mutation cosegregates with a rare connexin40 genotype in familial atrial standstill. *Circ Res* **2003**, *92*, 14-22.
144. Casini, S.; Tan, H.L.; Bhuiyan, Z.A.; Bezzina, C.R.; Barnett, P.; Cerbai, E.; Mugelli, A.; Wilde, A.A.; Veldkamp, M.W. Characterization of a novel SCN5A mutation associated with Brugada syndrome reveals involvement of DIIIS4-S5 linker in slow inactivation. *Cardiovascular research* **2007**, *76*, 418-429.
145. Turker, I.; Makiyama, T.; Vatta, M.; Itoh, H.; Ueyama, T.; Shimizu, A.; Ai, T.; Horie, M. A Novel SCN5A Mutation Associated with Drug Induced Brugada Type ECG. *PloS one* **2016**, *11*, e0161872.
146. Samani, K.; Wu, G.; Ai, T.; Shuraih, M.; Mathuria, N.S.; Li, Z.; Sohma, Y.; Purevjav, E.; Xi, Y.; Towbin, J.A., et al. A novel SCN5A mutation V1340I in Brugada syndrome augmenting arrhythmias during febrile illness. *Heart rhythm* **2009**, *6*, 1318-1326.
147. Samani, K.; Ai, T.; Towbin, J.A.; Brugada, R.; Shuraih, M.; Xi, Y.; Wu, G.; Cheng, J.; Vatta, M. A nonsense SCN5A mutation associated with Brugada-type electrocardiogram and intraventricular conduction defects. *Pacing and clinical electrophysiology : PACE* **2009**, *32*, 1231-1236.
148. Kyndt, F.; Probst, V.; Potet, F.; Demolombe, S.; Chevallier, J.C.; Baro, I.; Moisan, J.P.; Boisseau, P.; Schott, J.J.; Escande, D., et al. Novel SCN5A mutation leading either to isolated cardiac conduction defect or Brugada syndrome in a large French family. *Circulation* **2001**, *104*, 3081-3086.
149. Tan, B.H.; Valdivia, C.R.; Song, C.; Makielski, J.C. Partial expression defect for the SCN5A missense mutation G1406R depends on splice variant background Q1077 and rescue by mexiletine. *Am J Physiol Heart Circ Physiol* **2006**, *291*, H1822-1828.

150. Xiong, Q.; Cao, L.; Hu, J.; Marian, A.J.; Hong, K. A rare loss-of-function SCN5A variant is associated with lidocaine-induced ventricular fibrillation. *Pharmacogenomics J* **2014**, *14*, 372-375.
151. Zhu, J.F.; Du, L.L.; Tian, Y.; Du, Y.M.; Zhang, L.; Zhou, T.; Tian, L.I. Novel heterozygous mutation c.4282G>T in the SCN5A gene in a family with Brugada syndrome. *Exp Ther Med* **2015**, *9*, 1639-1645.
152. Maury, P.; Moreau, A.; Hidden-Lucet, F.; Leenhardt, A.; Fressart, V.; Berthet, M.; Denjoy, I.; Bennamar, N.; Rollin, A.; Cardin, C., et al. Novel SCN5A mutations in two families with "Brugada-like" ST elevation in the inferior leads and conduction disturbances. *J Interv Card Electrophysiol* **2013**, *37*, 131-140.
153. Deschenes, I.; Baroudi, G.; Berthet, M.; Barde, I.; Chalvidan, T.; Denjoy, I.; Guicheney, P.; Chahine, M. Electrophysiological characterization of SCN5A mutations causing long QT (E1784K) and Brugada (R1512W and R1432G) syndromes. *Cardiovascular research* **2000**, *46*, 55-65.
154. Six, I.; Hermida, J.S.; Huang, H.; Gouas, L.; Fressart, V.; Benammar, N.; Hainque, B.; Denjoy, I.; Chahine, M.; Guicheney, P. The occurrence of Brugada syndrome and isolated cardiac conductive disease in the same family could be due to a single SCN5A mutation or to the accidental association of both diseases. *Europace : European pacing, arrhythmias, and cardiac electrophysiology : journal of the working groups on cardiac pacing, arrhythmias, and cardiac cellular electrophysiology of the European Society of Cardiology* **2008**, *10*, 79-85.
155. Zumhagen, S.; Veldkamp, M.W.; Stallmeyer, B.; Baartscheer, A.; Eckardt, L.; Paul, M.; Remme, C.A.; Bhuiyan, Z.A.; Bezzina, C.R.; Schulze-Bahr, E. A heterozygous deletion mutation in the cardiac sodium channel gene SCN5A with loss- and gain-of-function characteristics manifests as isolated conduction disease, without signs of Brugada or long QT syndrome. *PloS one* **2013**, *8*, e67963.
156. Zhang, Z.S.; Tranquillo, J.; Neplioueva, V.; Bursac, N.; Grant, A.O. Sodium channel kinetic changes that produce Brugada syndrome or progressive cardiac conduction system disease. *Am J Physiol Heart Circ Physiol* **2007**, *292*, H399-407.
157. Grant, A.O.; Carboni, M.P.; Neplioueva, V.; Starmer, C.F.; Memmi, M.; Napolitano, C.; Priori, S. Long QT syndrome, Brugada syndrome, and conduction system disease are linked to a single sodium channel mutation. *J Clin Invest* **2002**, *110*, 1201-1209.
158. Saber, S.; Amarouch, M.Y.; Fazelifar, A.F.; Haghjoo, M.; Emkanjoo, Z.; Alizadeh, A.; Houshmand, M.; Gavrilenko, A.V.; Abriel, H.; Zaklyazminskaya, E.V. Complex genetic background in a large family with Brugada syndrome. *Physiol Rep* **2015**, *3*.
159. Postema, P.G.; Mosterd, A.; Hofman, N.; Alders, M.; Wilde, A.A. Sodium channelopathies: do we really understand what's going on? *Journal of cardiovascular electrophysiology* **2011**, *22*, 590-593.
160. Bennett, P.B.; Yazawa, K.; Makita, N.; George, A.L., Jr. Molecular mechanism for an inherited cardiac arrhythmia. *Nature* **1995**, *376*, 683-685.
161. Wang, D.W.; Yazawa, K.; George, A.L., Jr.; Bennett, P.B. Characterization of human cardiac Na+ channel mutations in the congenital long QT syndrome. *Proc Natl Acad Sci U S A* **1996**, *93*, 13200-13205.
162. Chandra, R.; Starmer, C.F.; Grant, A.O. Multiple effects of KPQ deletion mutation on gating of human cardiac Na+ channels expressed in mammalian cells. *Am J Physiol* **1998**, *274*, H1643-1654.
163. Nagatomo, T.; Fan, Z.; Ye, B.; Tonkovich, G.S.; January, C.T.; Kyle, J.W.; Makielski, J.C. Temperature dependence of early and late currents in human cardiac wild-type and long Q-T DeltaKPQ Na+ channels. *Am J Physiol* **1998**, *275*, H2016-2024.
164. Makita, N.; Behr, E.; Shimizu, W.; Horie, M.; Sunami, A.; Crotti, L.; Schulze-Bahr, E.; Fukuhara, S.; Mochizuki, N.; Makiyama, T., et al. The E1784K mutation in SCN5A is associated with mixed clinical phenotype of type 3 long QT syndrome. *J Clin Invest* **2008**, *118*, 2219-2229.
165. Malan, D.; Friedrichs, S.; Fleischmann, B.K.; Sasse, P. Cardiomyocytes obtained from induced pluripotent stem cells with long-QT syndrome 3 recapitulate typical disease-specific features in vitro. *Circ Res* **2011**, *109*, 841-847.
166. Zheng, J.; Zhou, F.; Su, T.; Huang, L.; Wu, Y.; Yin, K.; Wu, Q.; Tang, S.; Makielski, J.C.; Cheng, J. The biophysical characterization of the first SCN5A mutation R1512W identified in Chinese sudden unexplained nocturnal death syndrome. *Medicine (Baltimore)* **2016**, *95*, e3836.
167. Yokoi, H.; Makita, N.; Sasaki, K.; Takagi, Y.; Okumura, Y.; Nishino, T.; Makiyama, T.; Kitabatake, A.; Horie, M.; Watanabe, I., et al. Double SCN5A mutation underlying asymptomatic Brugada syndrome. *Heart rhythm* **2005**, *2*, 285-292.

168. Liang, P.; Liu, W.L.; Hu, D.Y.; Li, C.L.; Tao, W.H.; Li, L. [Novel SCN5A gene mutations associated with Brugada syndrome: V95I, A1649V and delF1617]. *Zhonghua Xin Xue Guan Bing Za Zhi* **2006**, *34*, 616-619.
169. Chen, T.; Inoue, M.; Sheets, M.F. Reduced voltage dependence of inactivation in the SCN5A sodium channel mutation delF1617. *Am J Physiol Heart Circ Physiol* **2005**, *288*, H2666-2676.
170. Dumaine, R.; Towbin, J.A.; Brugada, P.; Vatta, M.; Nesterenko, D.V.; Nesterenko, V.V.; Brugada, J.; Brugada, R.; Antzelevitch, C. Ionic mechanisms responsible for the electrocardiographic phenotype of the Brugada syndrome are temperature dependent. *Circ Res* **1999**, *85*, 803-809.
171. Wang, D.W.; Makita, N.; Kitabatake, A.; Balser, J.R.; George, A.L., Jr. Enhanced Na(+) channel intermediate inactivation in Brugada syndrome. *Circ Res* **2000**, *87*, E37-43.
172. Baroudi, G.; Carbonneau, E.; Pouliot, V.; Chahine, M. SCN5A mutation (T1620M) causing Brugada syndrome exhibits different phenotypes when expressed in Xenopus oocytes and mammalian cells. *FEBS Lett* **2000**, *467*, 12-16.
173. Shirai, N.; Makita, N.; Sasaki, K.; Yokoi, H.; Sakuma, I.; Sakurada, H.; Akai, J.; Kimura, A.; Hiraoka, M.; Kitabatake, A. A mutant cardiac sodium channel with multiple biophysical defects associated with overlapping clinical features of Brugada syndrome and cardiac conduction disease. *Cardiovascular research* **2002**, *53*, 348-354.
174. Vilin, Y.Y.; Fujimoto, E.; Ruben, P.C. A novel mechanism associated with idiopathic ventricular fibrillation (IVF) mutations R1232W and T1620M in human cardiac sodium channels. *Pflugers Archiv : European journal of physiology* **2001**, *442*, 204-211.
175. Baroudi, G.; Acharfi, S.; Larouche, C.; Chahine, M. Expression and intracellular localization of an SCN5A double mutant R1232W/T1620M implicated in Brugada syndrome. *Circ Res* **2002**, *90*, E11-16.
176. Makita, N.; Mochizuki, N.; Tsutsui, H. Absence of a trafficking defect in R1232W/T1620M, a double SCN5A mutant responsible for Brugada syndrome. *Circ J* **2008**, *72*, 1018-1019.
177. Zeng, Z.; Zhou, J.; Hou, Y.; Liang, X.; Zhang, Z.; Xu, X.; Xie, Q.; Li, W.; Huang, Z. Electrophysiological characteristics of a SCN5A voltage sensors mutation R1629Q associated with Brugada syndrome. *PloS one* **2013**, *8*, e78382.
178. Nakajima, T.; Kaneko, Y.; Saito, A.; Ota, M.; Iijima, T.; Kurabayashi, M. Enhanced fast-inactivated state stability of cardiac sodium channels by a novel voltage sensor SCN5A mutation, R1632C, as a cause of atypical Brugada syndrome. *Heart rhythm* **2015**, *12*, 2296-2304.
179. Tang, L.; Chehab, N.; Wieland, S.J.; Kallen, R.G. Glutamine substitution at alanine1649 in the S4-S5 cytoplasmic loop of domain 4 removes the voltage sensitivity of fast inactivation in the human heart sodium channel. *J Gen Physiol* **1998**, *111*, 639-652.
180. Kranjcec, D.; Bergovec, M.; Rougier, J.S.; Raguz, M.; Pavlovic, S.; Jespersen, T.; Castella, V.; Keller, D.I.; Abriel, H. Brugada syndrome unmasked by accidental inhalation of gasoline vapors. *Pacing and clinical electrophysiology : PACE* **2007**, *30*, 1294-1298.
181. Nunez, L.; Barana, A.; Amoros, I.; de la Fuente, M.G.; Dolz-Gaiton, P.; Gomez, R.; Rodriguez-Garcia, I.; Mosquera, I.; Monserrat, L.; Delpon, E., et al. p.D1690N Nav1.5 rescues p.G1748D mutation gating defects in a compound heterozygous Brugada syndrome patient. *Heart rhythm* **2013**, *10*, 264-272.
182. Zeng, Z.; Xie, Q.; Huang, Y.; Zhao, Y.; Li, W.; Huang, Z. p.D1690N sodium voltage-gated channel alpha subunit 5 mutation reduced sodium current density and is associated with Brugada syndrome. *Mol Med Rep* **2016**, *13*, 5216-5222.
183. Chen, Y.Y.; Liu, S.R.; Xie, L.Z.; Zhu, T.Y.; Chen, Y.Z.; Deng, X.J.; Meng, S.R.; Peng, J. [Functional analysis of a novel SCN5A mutation G1712C identified in Brugada syndrome]. *Nan Fang Yi Ke Da Xue Xue Bao* **2016**, *37*, 256-260.
184. Amin, A.S.; Verkerk, A.O.; Bhuiyan, Z.A.; Wilde, A.A.; Tan, H.L. Novel Brugada syndrome-causing mutation in ion-conducting pore of cardiac Na+ channel does not affect ion selectivity properties. *Acta Physiol Scand* **2005**, *185*, 291-301.
185. Vernooy, K.; Sicouri, S.; Dumaine, R.; Hong, K.; Oliva, A.; Burashnikov, E.; Timmermans, C.; Delhaas, T.; Crijns, H.J.; Antzelevitch, C., et al. Genetic and biophysical basis for bupivacaine-induced ST segment elevation and VT/VF. Anesthesia unmasked Brugada syndrome. *Heart rhythm* **2006**, *3*, 1074-1078.

186. Valdivia, C.R.; Tester, D.J.; Rok, B.A.; Porter, C.B.; Munger, T.M.; Jahangir, A.; Makielski, J.C.; Ackerman, M.J. A trafficking defective, Brugada syndrome-causing SCN5A mutation rescued by drugs. *Cardiovascular research* **2004**, *62*, 53-62.
187. Nakajima, T.; Kaneko, Y.; Saito, A.; Irie, T.; Tange, S.; Iso, T.; Kurabayashi, M. Identification of six novel SCN5A mutations in Japanese patients with Brugada syndrome. *Int Heart J* **2011**, *52*, 27-31.
188. Wei, J.; Wang, D.W.; Alings, M.; Fish, F.; Wathen, M.; Roden, D.M.; George, A.L., Jr. Congenital long-QT syndrome caused by a novel mutation in a conserved acidic domain of the cardiac Na+ channel. *Circulation* **1999**, *99*, 3165-3171.
189. Veltmann, C.; Barajas-Martinez, H.; Wolpert, C.; Borggrefe, M.; Schimpf, R.; Pfeiffer, R.; Caceres, G.; Burashnikov, E.; Antzelevitch, C.; Hu, D. Further Insights in the Most Common SCN5A Mutation Causing Overlapping Phenotype of Long QT Syndrome, Brugada Syndrome, and Conduction Defect. *Journal of the American Heart Association* **2016**, *5*.
190. Hofman-Bang, J.; Behr, E.R.; Hedley, P.; Tfelt-Hansen, J.; Kanters, J.K.; Haunsoe, S.; McKenna, W.J.; Christiansen, M. High-efficiency multiplex capillary electrophoresis single strand conformation polymorphism (multi-CE-SSCP) mutation screening of SCN5A: a rapid genetic approach to cardiac arrhythmia. *Clin Genet* **2006**, *69*, 504-511.
191. Kanters, J.K.; Yuan, L.; Hedley, P.L.; Stoevring, B.; Jons, C.; Bloch Thomsen, P.E.; Grunnet, M.; Christiansen, M.; Jespersen, T. Flecainide provocation reveals concealed brugada syndrome in a long QT syndrome family with a novel L1786Q mutation in SCN5A. *Circ J* **2014**, *78*, 1136-1143.
192. Rivolta, I.; Abriel, H.; Tateyama, M.; Liu, H.; Memmi, M.; Vardas, P.; Napolitano, C.; Priori, S.G.; Kass, R.S. Inherited Brugada and long QT-3 syndrome mutations of a single residue of the cardiac sodium channel confer distinct channel and clinical phenotypes. *J Biol Chem* **2001**, *276*, 30623-30630.
193. Dolz-Gaiton, P.; Nunez, M.; Nunez, L.; Barana, A.; Amoros, I.; Matamoros, M.; Perez-Hernandez, M.; Gonzalez de la Fuente, M.; Alvarez-Lopez, M.; Macias-Ruiz, R., et al. Functional characterization of a novel frameshift mutation in the C-terminus of the Nav1.5 channel underlying a Brugada syndrome with variable expression in a Spanish family. *PloS one* **2013**, *8*, e81493.
194. Gando, I.; Morganstein, J.; Jana, K.; McDonald, T.V.; Tang, Y.; Coetzee, W.A. Infant sudden death: Mutations responsible for impaired Nav1.5 channel trafficking and function. *Pacing and clinical electrophysiology : PACE* **2017**, *40*, 703-712.
195. Petitprez, S.; Jespersen, T.; Pruvot, E.; Keller, D.I.; Corbaz, C.; Schlapfer, J.; Abriel, H.; Kucera, J.P. Analyses of a novel SCN5A mutation (C1850S): conduction vs. repolarization disorder hypotheses in the Brugada syndrome. *Cardiovascular research* **2008**, *78*, 494-504.
196. Wang, D.W.; Desai, R.R.; Crotti, L.; Arnestad, M.; Insolia, R.; Pedrazzini, M.; Ferrandi, C.; Vege, A.; Rognum, T.; Schwartz, P.J., et al. Cardiac sodium channel dysfunction in sudden infant death syndrome. *Circulation* **2007**, *115*, 368-376.
197. Bebarova, M.; O'Hara, T.; Geelen, J.L.; Jongbloed, R.J.; Timmermans, C.; Arens, Y.H.; Rodriguez, L.M.; Rudy, Y.; Volders, P.G. Subepicardial phase 0 block and discontinuous transmural conduction underlie right precordial ST-segment elevation by a SCN5A loss-of-function mutation. *Am J Physiol Heart Circ Physiol* **2008**, *295*, H48-58.

© 2019 by the authors. Submitted for possible open access publication under the terms and conditions of the Creative Commons Attribution (CC BY) license (http://creativecommons.org/licenses/by/4.0/).
